# Adverse outcomes for autistic people: an umbrella review of mental health, physical health, social and lifestyle domains

**DOI:** 10.3389/fpsyt.2026.1702822

**Published:** 2026-05-11

**Authors:** Blandine French, Victoria Newell, Gamze Nalbant, Hannah Wright, David Daley, Sarah Cassidy

**Affiliations:** 1 School of Psychology, University of Nottingham, Nottingham, United Kingdom; 2 Nottingham Trent University, Nottingham, United Kingdom

**Keywords:** adverse outcomes, autism, co-occurring conditions, impact, umbrella review

## Abstract

**Background:**

Autistic people experience an increased risk of a wide range of adverse outcomes across many domains, impacting all aspects of daily life. While individual reviews have explored specific outcome areas, a comprehensive cross-domain synthesis is lacking. Because the evidence spans diverse outcome types and review designs, a narrative umbrella synthesis is needed to integrate findings across heterogeneous reviews and provide a broader systems-level overview.

**Methods:**

This umbrella review identified and synthesised published systematic, meta-analytic, and narrative reviews on the outcomes associated with autism. Five databases were searched from inception to June 2024. Dual screening and multi-reviewer data extraction were conducted, and methodological quality was appraised using established review tools. Findings were aggregated using inductive narrative synthesis, with domain grouping and thematic classification determined by consensus among reviewers.

**Results:**

Of 13,841 records identified, 134 reviews met inclusion criteria. Outcomes were grouped into three organisational domains: mental health, physical health, and social and lifestyle. The most consistently reported adverse outcomes included anxiety and mood disorders, suicidality and self-harm, eating and psychotic disorders, sleep problems, epilepsy, gastrointestinal and feeding difficulties, obesity, oral health problems, increased mortality risk, victimisation and bullying, reduced quality of life, loneliness, relationship difficulties, and unemployment. Evidence strength varied across outcomes, but elevated risk patterns were reported across multiple independent reviews.

**Discussion:**

Adverse outcomes for autistic people affect multiple areas of life. This umbrella synthesis identifies the principal outcome clusters supported by review-level evidence and highlights research, clinical practice, and service planning priorities.

**Systematic Review Registration:**

https://www.crd.york.ac.uk/prospero/, identifier CRD42023475389.

## Introduction

Autism is a lifelong neurodevelopmental condition characterised by distinct cognitive and behavioural profiles that influence social interactions, school and family life (1). Many autistic people^1^ experience differences with social communication and interaction, preference for routine and familiarity, and focused interests (2), where variations in sensory processing (3), emotion regulation (4) or motor and social skills (5) are also common. Furthermore, many autistic people experience co-occurring neurodevelopmental and psychiatric conditions (6). In a recent review, Barlattani and colleagues (7) established eight categories based on psychiatric co-occurrence in autism: attention deficit hyperactivity disorders; anxiety disorders; personality disorders; repetitive behaviours and obsessive-compulsive disorders; sleep disorders; mood disorders; Tourette syndrome and tic disorder studies; as well as feeding and eating disorders. Other mental health conditions have been linked to autism (8), including depression, bipolar disorder, schizophrenia, conduct disorders, and sleep-wake disorders. Likewise, autistic people are at increased risk of anxiety/depression, self-harm and drug use (9).

Beyond psychiatric co-occurring conditions, many other long-term adverse outcomes are experienced by autistic people, encompassing health-related issues (e.g., obesity), societal issues (e.g., increased risk of contact with the criminal justice system) or daily functions (e.g., difficulties at work). For example, autistic people frequently face significant academic and educational challenges (10), overrepresentation in the criminal justice system (11), pervasive social exclusion (12), driving accidents (13), unemployment (14) and suicide (15). Several reviews and population studies have investigated specific links between autism and related difficulties (7, 8, 16–23). Howlin and Magiati (18) showed autistic children had an increased risk of a range of negative outcomes in adulthood, such as poor mental health, decreased social functioning, and lower life satisfaction. Additionally, autistic adults are at a higher risk for substance abuse, suicide, mortality and lower quality of life. In terms of physical health, autistic people are more likely to exhibit epilepsy, macrocephaly, hydrocephalus, cerebral palsy, migraine/headache, sleep, atopy, obesity and gastrointestinal features (23–25). These conditions represent adverse outcomes in their own right and may also interact with other life domains, contributing to cumulative challenges across the lifespan.

Numerous individual studies have identified a range of different long-term outcomes experienced by autistic people, and a few reviews have focused on synthesising specific outcomes (such as psychiatric co-occurring conditions or health). However, no research has yet synthesised the broader evidence to identify the full range of challenges and inequalities that autistic people may encounter across the lifespan. A comprehensive synthesis is therefore crucial to understanding the broader experiences of autistic people, informing holistic support strategies, highlighting unmet needs, and advocating for systemic change. While existing reviews provide valuable insights, they do not capture the breadth or the interaction amongst these outcomes needed to inform effective policy and practice.

To address this gap, the present umbrella review synthesises review-level evidence on adverse outcomes experienced by autistic people across the lifespan. These outcomes occur within and are shaped by multiple interacting systems, including healthcare, education, social care, employment, and justice contexts, where access, accommodation, and support can vary widely. A cross-domain synthesis is therefore needed to understand not only the range of reported outcomes, but also their implications for service planning and system-level responses. Because the literature spans diverse outcome types and review designs, we used a narrative umbrella synthesis approach to integrate heterogeneous evidence.

The review was guided by the following questions: (1) What adverse outcomes experienced by autistic people have been identified across published reviews? and (2) How consistently are these outcomes supported across the review literature? Specifically, this review aims to systematically synthesise evidence on adverse outcomes reported across mental health, physical health, and social domains to highlight areas of consistent evidence, identify knowledge gaps, and consider implications for support and systemic change throughout the lives of autistic people. To ensure interpretability across a large and diverse evidence base, outcome domains were derived through inductive narrative synthesis during the aggregation process, rather than specified *a priori*, and were organised into three broad groupings: mental health, physical health, and social and lifestyle.

## Methods

This umbrella review was conducted in accordance with the Preferred Reporting Items for Systematic Reviews and Meta-analysis Protocols (PRISMA-P) guidelines (26) and the Joanna Briggs Institute (JBI) Manual for Umbrella Reviews (27).

A protocol for this review is registered with the International Prospective Register of Systematic Reviews (PROSPERO; CRD42023475389). The search was conducted on the 24th of June 2024.

### Inclusion criteria

#### Type of population

Eligible reviews included individuals (adults and children) who met the review authors’ criteria for, or had received a diagnosis of, autism. This criterion was based on a variety of methods, including meeting DSM or ICD criteria, self-report, or achieving a specified cut-off on a validated measure. If reviews included multiple groups, such as ADHD and autistic people, autism findings were extracted and reported separately if possible.

#### Type of phenomenon of interest

This review examines the adverse outcomes reported in the literature as being experienced by autistic people. Within the context of this review, outcomes are defined as disparities or inequalities experienced by autistic people in everyday life, encompassing effects for the individual, their environments (e.g., work, school, friendships), their families, and others around them. We acknowledge that many adverse outcomes are likely influenced by shared vulnerabilities, co-occurring conditions, and, critically, environmental factors and systemic barriers (e.g., lack of support, stigma, unaccommodating environments) rather than being solely inherent to autism itself. The precise ways in which impact is defined and measured differ between studies in this field. This review, therefore, aimed to capture broader concepts such as risks, effects, outcomes, and consequences to reflect the complexity of autistic people lived experiences and challenges.

#### Context

The included reviews were conducted in various settings and took an international perspective. The review period was unrestricted, encompassing all publications from inception through June 2024.

#### Type of studies

This review included only published quantitative and qualitative reviews and considered studies that examine the impacts, long-term outcomes, or adverse outcomes experienced by autistic people (including, but not limited to, narrative reviews, systematic reviews with or without meta-analysis, or scoping reviews). Only studies published in peer-reviewed publications were considered.

### Exclusion criteria

Unpublished and grey literature, as well as non-peer-reviewed publications, were excluded. Reviews were also excluded if they did not clearly specify the neurodevelopmental condition examined, did not focus on diagnosed individuals, or did not explicitly refer to autism. Reviews examining impacts primarily linked to other neurodevelopmental conditions with common co-occurring presentations (e.g., ADHD, dyspraxia) were excluded. Only review articles were eligible; primary studies were not included. Reviews focused on prevalence, assessment, interventions, management, risk factors, treatment, or treatment outcomes were excluded, as were those examining biological features (e.g., brain correlates, genetics, biological mechanisms, cognitive testing, executive or motor functioning). Finally, reviews were excluded if they did not report a direct association between autism diagnostic status and adverse outcomes, or if effects could not be disaggregated from other linked factors (e.g., autism, cognition, and eating).

To ensure a broad synthesis across diverse global contexts, reviews reporting findings exclusively from a single country or region were excluded. The aim was to identify outcome patterns observable across international settings rather than those limited to country-specific contexts.

### Search strategy

The full search terms for health risks and strategy are detailed below for Medline and were adapted for other databases:

1. exp Autistic Disorder/.
2. (ASC or ASD or Asperg* or Autis* or “high functioning” or “pervasive developmental disorder*” or PDD or HFA).tw,kw,kf.
3. 1 or 2.
4. (risk* or impact or correlation or long-term outcome* or longterm outcome* or association).tw,kw,kf.
5. exp risk/.
6. 4 or 5.
7. 3 and 6.
8. exp “Wounds and Injuries”/or exp Body Weight/or exp Health/or exp Cardiorespiratory Fitness/or exp Men’s Health/or exp Occupational Health/or exp Fitness/or exp Women’s Health/.
9. (physical health or physical fitness or health status or body weight or obesity or injur*).tw,kw,kf.
10. (cardiovascular or respiratory or metabolic or endocrine or gastrointestinal).tw,kw,kf.
11. 8 or 9 or 10.
12. 7 and 11.
13. exp Mental Health/or exp Mental Disorders/.
14. (mental adj2 (health or wellbeing or well-being or disorder* or disease* or illness)).tw,kw,kf.
15. ((psychiatric or psychological) adj2 (comorbid* or co-morbid* or condition* or illness or disorder* or health)).tw,kw,kf.
16. 13 or 14 or 15.
17. 7 and 16.
18. exp Meta-Analysis as Topic/.
19. exp meta analysis/.
20. (meta analy$ or metaanaly$ or “meta synthesis” or metasynthesis).tw.
21. exp “systematic review”/.
22. (systematic adj (review$1 or overview$1)).tw.
23. exp Review Literature as Topic/.
24. (“evidence based review” or “comprehensive review*” or “quantitative* review*” or “quantitative* overview*” or “quantitative* synthesis*” or “qualitative review*” or “qualitative overview*” or “qualitative synthesis” or “narrative review*” or “realist review*” or “umbrella review*” or “review of reviews”).tw.
25. 18 or 19 or 20 or 21 or 22 or 23 or 24.
26. (“selection criteria” or “data extraction”).ab.
27. exp “review”/.
28. 26 and 27.
29. 25 or 28.
30. 12 and 29.
31. 17 and 29.

Five databases (PsycINFO, Embase, Scopus, Medline, ERIC) were searched. The search covered all records from database inception to 24 June 2024, when the final searches were conducted. Following this search and duplicate removal, a preliminary analysis was conducted of the subject headings (MeSH) and text words related to autism and adverse outcomes. Examples included MeSH terms such as *Autistic Disorder* and *Risk*, and text words including “autis*” and “adverse outcomes”. PROSPERO was checked for ongoing or published systematic reviews on the subject. Although hand searching was not a strong component of our planned search strategy, the reference lists of all papers that met the inclusion criteria were hand-searched to identify any additional reviews.

### Study selection

**Figure 1:**
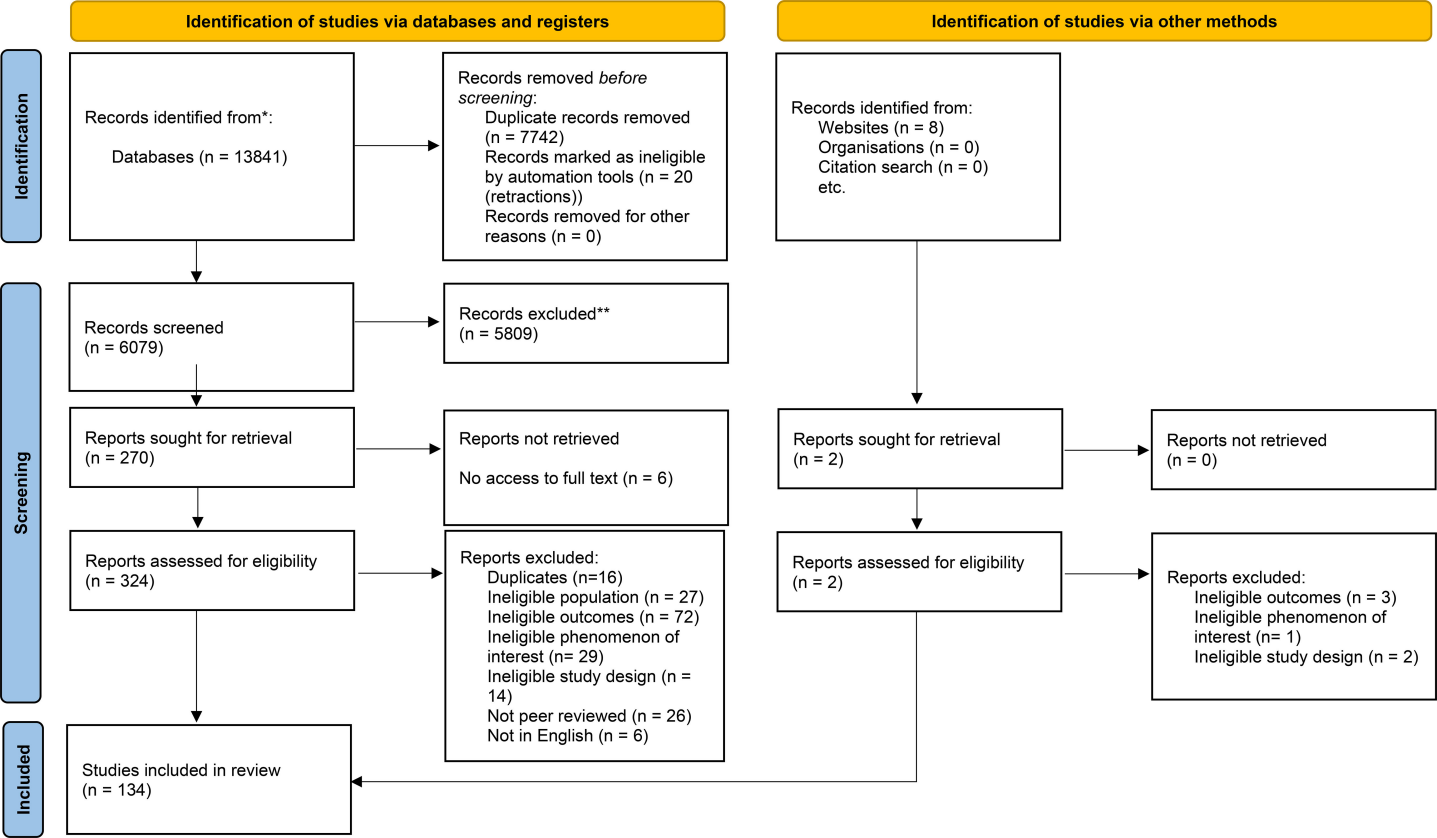
Study selection PRISMA flow diagram.

Following the search, all identified citations were deduplicated and uploaded to the reference management software (Rayyan). Two review authors (BF and GN) independently screened the titles and abstracts to determine whether they met the search inclusion criteria. Complete reports were obtained for all titles that met the inclusion criteria. The same two review authors independently screened and assessed the full-text reports against the inclusion criteria. Studies that did not meet the inclusion criteria were excluded, and a record of the exclusion reasons was maintained. The study selection process is presented below (**Figure 1**).

### Data extraction and outcomes

#### Data extraction

Three reviewers (VN, SC, and BF) independently extracted qualitative and quantitative data from the included studies and cross-checked them for accuracy. Disagreements between reviewers at screening, extraction, and quality appraisal stages were resolved through discussion and consensus. Where consensus could not be reached, a third reviewer adjudicated. Reasons for inclusion/exclusion decisions, as well as adjudications, were documented.

#### Outcomes

The primary outcome is the synthesis of impacts and adverse outcomes experienced by autistic people. Multiple types of factors reported in the selected studies were evaluated, such as mental health, societal factors (divorce, imprisonment, etc.) and health factors (suicide, drug abuse, etc.). These factors were grouped into domains within the synthesis phase.

### Data synthesis

Qualitative and quantitative findings were aggregated into a narrative synthesis. Findings were aggregated into organisational domains to generate a coherent synthesis framework. Three reviewers conducted the synthesis in sequence: two reviewers developed the synthesis (SC and VN), and the third (BF) checked the findings. Any disagreement was discussed and/or mediated by an additional reviewer. The themes were identified through an inductive narrative synthesis (28), where key concepts and patterns were developed directly from the included reviews. Domains and themes were generated inductively through an iterative narrative synthesis process involving multiple reviewers, repeated cross-review comparison, draft domain grouping, and consensus discussions to refine classifications. The resulting domains function as organisational categories to support synthesis across a large and heterogeneous evidence base rather than as mutually exclusive conceptual groupings. Where constructs plausibly spanned more than one domain, classification was based on the primary outcome framing and emphasis used in the source review. Domain and theme decisions were reviewed and refined through multi-reviewer consensus and comparison with previously published umbrella (29) and narrative synthesis frameworks to maximise conceptual coherence and interpretability, and to acknowledge expected conceptual overlap in the narrative synthesis.

### Assessment of methodological quality

Following mixed methods review guidelines (30), two review authors (HW and BF) independently critically appraised all selected studies for methodological quality. One hundred and sixteen of the studies were systematic reviews (with or without meta-analysis) and were assessed using the Joanna Briggs Institute Tool for Systematic Reviews (31). The remaining 18 literature or narrative reviews were scored using the Scale for the Assessment of Narrative Review Articles (SANRA, 67), as it was not appropriate to score them using the systematic review checklist. Any disagreements between reviewers were resolved through discussion and/or consultation with a third reviewer. The quality results are reported in the main study table (**Table 1**). The quality rating of each study did not affect its inclusion; all studies that met the inclusion criteria were submitted to the data extraction and synthesis process. Methodological transparency and rigour were supported by protocol registration (PROSPERO), predefined extraction procedures, dual-review processes, and documentation of consensus decisions during synthesis and classification.

**Table 1:** Summary of review characteristics and quality assessment (Please refer to the original **Table 1** in Appendix 1 for full details).

Mental health			
Topic health	Number of reviews	Association	No association
Anxiety and mood disorders	14 reviews (7 anxiety; 1 social anxiety; 1 OCD; 9 depression, 2 bipolar disorder 10 mixed reviews (10 anxiety; 1 social anxiety; 8 OCD; 2 affective/mood; 10 depression; 8 bipolar)	5 reviews showed higher rates of anxiety, and 1 review showed OCD. 1 review found an association with social anxiety disorder. 7 reviews reported higher rates of depression. 2 reviews supported an association with bipolar disorder. All 10 mixed reviews found a high frequency of co-occurring mental health conditions in autism.	However, 2 of the reviews which reported higher rates of depression should be interpreted with caution. 1 review of both depression and anxiety suggested little evidence for an association. 1 review suggested mental health problems may not differ between autistic and non-autistic mothers.
Eating Disorders	4 reviews (2 anorexia nervosa) 7 mixed reviews	All reviews indicated an association with eating disorders, 2 of which focused on anorexia nervosa. All 7 mixed reviews found a high frequency of co-occurring eating disorders in autism.	
Psychotic and personality disorders	9 reviews (2 schizophrenia; 3 psychosis; 1 paranoia, 2 BPD, 1 PTSD) 9 mixed reviews (8 schizophrenia/psychotic disorders; 1 psychosis, 1 PTSD, 1 trauma and stress; 2 personality disorders; 1 BPD)	All reviews showed an association between schizophrenia, psychosis or paranoia. 2 out of 3 reviews found increased prevalence of BPD or PTSD All mixed reviews found a high frequency of co-occurring psychotic and personality disorders in autism.	1 out of 3 reviews did not find an increased prevalence of BPD
Substance misuse	3 reviews 5 mixed reviews	2 reviews demonstrated an increased risk of substance misuse. All 5 mixed reviews found a high frequency of co-occurring substance use in autism.	1 out of 3 reviews noted limited evidence for an association and that rates vary widely.
Suicide and self-harm	12 reviews (8 suicide; 2 self-harm; 2 intent undetermined) 2 mixed reviews (2 suicide)	10 out of 12 reviews showed an association with suicide or self-harm. Both mixed reviews found a high frequency of co-occurring suicide in autism	1 review implied the rate of suicidal behaviours is similar to that of the non-autistic population. 1 review noted insufficient data to determine rates of self-injury.

## Results

### Study selection

In total, 134 reviews published between 2013 and 2024 met the inclusion criteria. These included systematic reviews, meta-analyses, narrative reviews, rapid reviews, and scoping reviews. Study characteristics and corresponding review domains are presented in Appendix 1. The reviews represented a broad international context. Across the 134 included reviews, reporting of autism diagnostic ascertainment varied. Sixty-six reviews explicitly required a formal clinical diagnosis based on DSM, ICD, or clinician-confirmed diagnosis using established assessment measures. Five reviews included samples defined by either formal clinical diagnosis or self-reported diagnosis within their eligibility criteria. In contrast, 53 reviews did not explicitly state whether a formal diagnosis based on established criteria was required, referring more broadly to autistic individuals without clarifying diagnostic procedures.

### Data methodological quality

Study quality ratings are reported in **Table 1**. Following JBI guidance and Kmet, Lee and Cook’s criteria (32), systematic reviews were scored from 0–1 and categorised as poor (0–0.44), moderate (0.45–0.69), or good (0.70–1.00). The same thresholds were applied to SANRA scores for narrative reviews to ensure consistent reporting, although SANRA is typically scored out of 12. Reviews assessed using SANRA are marked in **Table 1**. Inter-rater agreement was 90%, with discrepancies resolved through discussion. Overall, most studies were of good methodological quality: 114 (85%) were rated good, 18 (13.5%) moderate, and 2 (1.5%) poor.

### Data extraction and summary of results

Findings were synthesised across three organisational domains: mental health, physical health, and social and lifestyle. Some sub-themes (e.g., identity, body image, quality of life) are inherently cross-cutting and may span psychological and social processes; domains are therefore presented as organisational anchors rather than mutually exclusive categories. Wide prevalence ranges reflect substantial heterogeneity in study design, sampling, and measurement across underlying reviews. Conclusions are therefore based on the consistency of the association rather than on pooled prevalence estimates.

#### Mental health (50 reviews, including 10 mixed reviews covering multiple domains)

The mental health domain examined anxiety and mood disorders, eating disorders, psychotic and personality disorders, substance misuse, and suicide and self-harm. **Table 1** summarises the mental health studies.

##### Anxiety and mood disorders (n=14)

Fourteen reviews (16, 33–38, 38–44) and ten mixed reviews (7, 8, 17, 18, 20–22, 45–47) examined anxiety and mood disorders. Across meta-analyses, autistic children and adolescents showed significantly higher prevalence of anxiety disorders (e.g., 39.6% for at least one anxiety disorder), particularly specific phobia (29.8%), OCD (17.4%), and social anxiety disorder (16.6%) compared to non-autistic peers (41, 44). Systematic reviews (36–39) also reported higher rates of depression, although prevalence estimates varied widely (1%–83.3%), partly reflecting differences in measurement tools and study populations. Two reviews reported an association with bipolar disorder in autistic adults, with prevalence ranging from 6% to 21.4% (37, 38).

Across mixed-topic reviews, prevalence estimates varied substantially (e.g., anxiety 1.47%–54% in narrative reviews; 11.1%–35% in meta-analyses) (8, 20, 22, 47). OCD ranged from 2%–33% in narrative reviews and 1.8%–9% in meta-analyses (8, 22, 47). Mood disorder prevalence ranged from 4.4% to 37% (17), whereas meta-analyses demonstrated a range of 18.8% to 19% (20, 47). Depressive disorder prevalence ranged from 0%–74.8% in reviews and 2.7%–18% in meta-analyses, with higher rates reported in autistic adults compared to children and adolescents (7, 16, 18, 48) and 2.7 - 18% in meta-analyses (8, 22, 47), where it was also higher in autistic adults (34%) vs children/adolescents. Bipolar disorder ranged from 1%–25% in reviews (7, 17, 47) and 2%–7% in meta-analyses (8, 22, 47).

##### Eating disorders (n=4)

Four reviews explored autism and eating disorders, including anorexia nervosa, bulimia nervosa, and binge eating disorder (49–52). Evidence indicates an overrepresentation of autistic traits in anorexia nervosa populations, with 8.8%–24.5% meeting criteria for possible autism (49, 52). Approximately 4.7% of individuals with an eating disorder (50) were reported to have a co-occurring autism diagnosis. Despite evidence of symptomatic overlap, authors noted challenges in distinguishing whether shared features (e.g., rigidity, sensory sensitivities, restricted eating) reflect autistic characteristics, eating disorder pathology, or co-occurring conditions such as depression.

Feeding and eating disorders were also included in seven reviews of multiple topics, supporting their high co-occurrence with autism (7, 17, 20, 21, 45–47). Reviews demonstrated prevalence rates of 1.4 – 7.9%, with anorexia nervosa being the most common subtype, followed by bulimia nervosa and binge eating disorder (7, 17, 20, 45).

##### Psychotic and personality disorders (n=9)

Nine reviews examined psychotic and personality disorders (53–58). Autistic people were over three times more likely to have schizophrenia than controls (OR = 3.55) (57). Meta-analyses reported a pooled prevalence of psychosis in autistic adults of 9.4%, and psychotic experiences in autism of 24% (56). Elevated paranoia was also reported (53).

Prevalence estimates in mixed-topic reviews ranged widely (0%–67% in narrative reviews; 4%–11.8% in meta-analyses) (8, 20, 47).

Evidence for Borderline Personality Disorder (BPD) was mixed (59–61). One review reported higher autistic traits in BPD populations (59), whereas a meta-analysis found pooled prevalence similar to the general population (BPD in autism 4%; autism in BPD 3%) (60). PTSD prevalence in autistic youth was similar to or higher than population estimates, though many samples were treatment-seeking (61).

##### Substance misuse (n=3)

Three reviews investigated substance misuse (62–64). Evidence suggests increased risk in autistic adults, with prevalence estimates ranging from 1.3%–36%, though findings were less consistent in younger samples (63). Mixed-topic reviews reported prevalence between 0.7%–36% in narrative reviews (17, 18) and 5% - 8.3% in meta-analyses where alcohol abuse/dependence disorder was the most frequent (20, 47).

##### Suicide and self-harm (n=11)

Eleven reviews examined suicidality and self-harm (65–75). Multiple high-quality reviews demonstrate an increased risk of suicidal ideation, suicide attempts, and suicide death (69, 70, 73). Autistic youth showed higher prevalence of suicidal ideation (25.2%), attempts (8.3%), and suicide deaths (0.2%) compared to non-autistic peers. Self-harm prevalence reached 42% in some reviews (67, 74). Mixed reviews reported suicidal ideation rates of 10.9%–66% and suicidal behaviours (plans or attempts) 1%–35% (18, 19). Risk factors included peer victimisation, co-occurring depression, and socioeconomic disadvantage (69, 70).

#### Physical health (63 reviews, including 6 mixed reviews)

The physical health domain included oral health; atopic dermatitis; diabetes; asthma; epilepsy; sleep; unhealthy weight/obesity; gastrointestinal and feeding problems; vision and/or hearing impairment/loss; mortality; and other conditions. **Table 2** summarises the physical health studies.

**Table 2:** Physical health outcomes.

Physical health			
Health area	Number of reviews	Association	No association
Oral health	7 (2 bruxism, 4 plaque index, 6 gingivitis index/periodontal disease, 5 dental caries)	Both meta-analyses showed increased prevalence of bruxism in autistic children and adolescents. All three meta-analyses and all studies in a systematic review showed significantly increased plaque index in autistic children and adolescents. 2/4 meta-analyses and all studies included in a systematic review showed increased gingivitis index/periodontal disease in autistic children and adolescents. 3/4 meta-analyses and some (not all) studies included in a systematic review showed evidence of increased risk of dental caries in autistic children and adolescents.	
Atopic dermatitis	3	All three meta-analyses showed a significant association between AD and autism.	
Diabetes	3	1/2 of the meta-analyses found an increased risk of diabetes in autistic people. Only 4/15 studies included in a systematic review found an association between autism and diabetes. Higher-quality studies that controlled for confounding factors did not find an association.	
Asthma	3	1/3 meta-analyses showed increased prevalence of asthma in autistic children and adolescents compared to controls.	
Epilepsy	9	All reviews showed increased risk of epilepsy in autistic people.	
Sleep	8 (3 sleep disorders/problems, 3 objective measures of specific sleep problems, 3 subjective measures of specific sleep problems, 1 impact on autistic traits and behaviour)	All three reviews showed increased prevalence of overall sleep disorders and sleep problems in autistic people. All three reviews found increased prevalence of objective measures of specific sleep problems. Mixed evidence of increased prevalence of some subjective measures of specific sleep problems.	
Unhealthy weight/obesity	6	All six reviews showed consistent evidence of increased prevalence of obesity in autistic people.	
Gastrointestinal problems (GI) and feeding problems	12 (8 GI problems, 2 GI disease, 2 celiac disease) 9 (2 feeding problems + nutritional intake, 5 food selectivity, 2 food allergies and intolerance).	All 8 reviews showed increased prevalence of GI problems in autistic people. Both reviews showed an increased risk of GI diseases. Both reviews showed no evidence for increased prevalence of celiac disease in autistic people. Two reviews showed increased prevalence of feeding problems associated with nutritional deficiencies. 3/5 reviews found an association between food selectivity and autism. Both reviews found an association between food allergies and intolerance and autism in children.	
Vision/hearing impairment/loss	5 (4 hearing, 4 vision)	4 reviews showed mixed evidence of increased prevalence of hearing impairment/loss in autistic people. 1/4 reviews evidence of increased prevalence of vision impairment in autistic people.	
Mortality	3	All three reviews showed increased risk of mortality in autistic people compared to the general population.	
Other	5 (1 incontinence, 1 catatonia, 1 congenital health disease, 1 neurological disorder, 1 allergy/autoimmune disease).	Each study found increased prevalence/risk of the physical health condition in autistic people.	

##### Oral health (n=7)

Seven reviews explored oral health in autistic people, with a majority (6/7) focused on adolescents and/or children (76–82). Most reviews indicated an increased plaque index (77, 78, 81, 82) and bruxism (teeth grinding) (79, 80, 82) in autistic children and adolescents. However, findings for gingivitis, periodontal disease (77, 78, 82), and dental caries were mixed (78), with some meta-analyses showing increased prevalence while others did not. For example, a high pooled prevalence of clinically observed bruxism (57.5%) was found in autistic children and adolescents (79).

##### Atopic dermatitis (n=3)

All three meta-analyses reported a significant association between autism and atopic dermatitis, with a higher pooled prevalence compared to controls (83–85).

##### Diabetes (n=3)

Three reviews showed mixed evidence of increased risk of diabetes in autistic people (86–88). Higher-quality studies controlling for confounding factors often did not find significant associations, suggesting age, obesity, medication use, and metabolic conditions may account for observed differences (88). One meta-analysis of 34 studies, however, showed a significantly higher pooled prevalence of diabetes in autistic people despite high study heterogeneity, with a significantly higher risk in autistic children compared to adults (86).

##### Asthma (n=3)

Three meta-analyses showed mixed evidence for increased risk of asthma in autistic children and adolescents (89–91). Two meta-analyses (89, 91) found no significant association between asthma and autism, while another showed a higher prevalence of asthma in autistic children and adolescents compared to controls (90).

##### Epilepsy (n=9)

Nine reviews consistently showed an increased risk of epilepsy in autistic people (16, 18, 19, 21, 24, 47, 92). All reviews reported increased epilepsy prevalence in autistic people (2.8%–43.75%). A meta-analysis reported a pooled prevalence of 7% in children and 19% in adults (93).

##### Sleep (n=8)

Eight reviews showed an increased prevalence of sleep difficulties in autistic people (21, 24, 94–99). All reviews reported increased prevalence of sleep problems. Objective measures indicated increased sleep-disordered breathing, more frequent awakenings at night, longer sleep onset latency, and reduced sleep duration. The prevalence of insomnia in adults was 58.98% (98).

##### Unhealthy weight/obesity (n=6)

Six reviews reported an increased risk of obesity across the lifespan (21, 24, 100–105). Autistic children had 41.1% greater risk of obesity compared to non-autistic peers (102). Evidence for being underweight was mixed (101, 105).

##### Gastrointestinal and feeding problems (n=12 for GI; n=9 for feeding)

Twelve reviews reported increased gastrointestinal symptoms, most commonly abdominal pain, constipation, diarrhoea, and functional bowel disturbances (16, 19, 21, 47, 106–113). Meta-analyses indicate a significantly increased risk of gastrointestinal problems in autistic children (33%-55%) compared to the general population (106, 109, 111), with abdominal pain, constipation, and diarrhoea being the most reported issues (19, 47, 107, 108, 111). Associations were reported for eosinophilic gastrointestinal disease (113) and inflammatory bowel disease (including ulcerative colitis and Crohn’s disease) (110), but not celiac disease (47, 112).

Regarding feeding problems, two reviews explored these in autistic infants and children, finding an increased risk of feeding problems associated with nutritional deficiencies (114, 115). Five reviews on food selectivity found an association between food selectivity and autism, despite varying definitions (107, 116–119). Two reviews explored food allergies and intolerance, finding autistic children were over-represented in those with food hypersensitivity (107, 120).

##### Vision and/or hearing impairment/loss (n=5)

Five reviews on vision and/or hearing impairment/loss in autistic people revealed mixed results (16, 21, 24, 121, 122). Evidence for increased vision or hearing impairment was mixed and highly heterogeneous.

##### Mortality (n=3)

Three reviews showed an increased risk of mortality in autistic people (18, 92, 123), at least twice that of the general population (18). Risk factors for increased mortality include co-occurring intellectual disability, epilepsy, and female sex (92).

##### Other physical health conditions (n=5)

Higher prevalence of incontinence, catatonia (10.4%), congenital heart disease, and neurological conditions (e.g., macrocephaly, cerebral palsy) were reported (23, 124–126). Evidence for allergy and autoimmune conditions was mixed (19, 24).

#### Social and lifestyle (29 reviews, including 3 on multiple factors)

The social and lifestyle domain explored outcomes experienced by autistic people related to body image and identity; bullying, victimisation and violence; gaming and internet use; offending and criminality; quality of life; relationships, social connection and sexual outcomes; as well as broader areas such as school absenteeism, driving, ageing, and unemployment. Several topics within this domain intersect with mental health constructs but are grouped here according to how the source reviews primarily framed outcomes. **Table 3** summarises all social and lifestyle studies.

**Table 3:** Social and lifestyle outcomes.

Social and lifestyle			
Topic area	Number of reviews	Association	No association
Body image and identity	3 reviews (1 body image; 2 TGNC identity) 2 mixed reviews (TGNC identity)	All reviews indicated an association with TGNC identities or body image. Both mixed reviews included one study showing an association with gender identity disorder.	
Bullying, victimisation and violence	6 reviews (3 bullying; 1 victimisation; 2 violence)	All reviews showed an association with several forms of bullying, victimisation and violence.	
Gaming and internet use	2 reviews (2 gaming; 1 internet)	Both reviews found an association between problematic gaming or internet use.	
Offending and criminality	4 reviews (1 delinquency; 1 criminality; 2 sexual offending) 1 mixed review (offending)	2 reviews suggest autistic people may be more likely to commit sexual offences, but more research is needed. 1 mixed review found 4 studies exploring offending as a challenging behaviour in autism.	1 review did not find an association with delinquent behaviour. 1 review did not find autistic people to be overrepresented by the criminal justice system.
Quality of life	2 reviews 1 mixed review	Both reviews demonstrated an association with a lower quality of life.	1 mixed review reports mixed evidence for quality-of-life outcomes.
Relationships, social connections, and sexual outcomes	6 reviews (2 friendships; 1 school social participation; 1 loneliness; 2 sexual outcomes)	All reviews found associations with various aspects of relationships, social connections, and sexual outcomes.	
Other	3 reviews (1 school; 1 driving; absenteeism; 1 ageing) 1 mixed review (employment)	1 review found an association with school absenteeism. 1 review suggested that autistic people experience driving-related challenges. 1 review indicates challenges associated with ageing in autism. 1 mixed review reports that autistic people are at an elevated risk of unemployment.	

##### Body image and identity (n=5)

Evidence suggests increased body image difficulties and a higher prevalence of transgender or gender non-conforming identities among autistic people (127–129). Emerging evidence suggests an association between autism and body image difficulties (125), including dissatisfaction with appearance, distress related to weight or shape, heightened self-consciousness, and body-related anxiety, particularly where sensory sensitivities or social pressures influence these experiences. There is consistent evidence of a high co-occurrence and overrepresentation of gender dysphoria and trans-identity in autistic people across various age ranges (47, 127).

##### Bullying, victimisation, and violence (n=6)

Six reviews demonstrated an association between autism and various forms of bullying, victimisation, and violence (128–133). Autistic people are at a significantly greater risk of bullying victimisation (with prevalence rates of 44% - 67%) and are twice as likely to be verbally bullied compared to their non-autistic peers (130, 132). Prevalence of broader victimisation in autistic people is high, including child abuse (16%), sexual victimisation (40%), and cyberbullying (13%) (129). Rates of interpersonal violence (emotional abuse, sexual and physical violence) may also be higher for autistic adults than in the general population (131).

##### Gaming and internet use (n=2)

Two reviews reported associations between autism and problematic gaming or internet use, defined as excessive use that impacts daily life (134, 135).

##### Offending and criminality (n=5)

Most reviews found no increased risk of general offending in autistic people (136, 137). However, a small number of studies explored sexual offending, with mixed findings and strong emphasis on contextual factors rather than autism itself (138).

##### Quality of life (n=2)

Two reviews found an association between quality of life and autism (139, 140). Evidence indicates that quality of life is lower for autistic people compared to non-autistic people (d = −0.96) (139).

##### Relationships, social connection, and sexual wellbeing (n=6)

All six reviews found an association of autism with multiple aspects of relationships, social connections, and sexual outcomes (141–146). Autistic children are reported to have fewer friends, lower quality friendships, and less friendship reciprocation compared to non-autistic peers (145). A meta-analysis also found significantly increased loneliness (143) and less social behaviour and social contact (141, 142).

##### Other social and lifestyle outcomes (n=4)

Four reviews examined additional social and lifestyle outcomes (40, 147–149), with some focusing on specific functional outcomes, such as driving, while others examined experiences of ageing in autism. Autistic children and adolescents are more often absent from school than their non-autistic peers, with bullying identified as a possible factor (150). Autistic drivers, particularly males, experience driving-related challenges, including slower reaction times and greater difficulty with tactical decisions (151). Autistic adults are at significant risk of unemployment, with the proportion of those reporting active employment ranging from 20% to 55.3% (31). Additionally, ageing in autism can present various challenges, including reduced quality of life due to co-occurring conditions, social isolation, and restrictions in accessing employment and residential options (152). Ageing is a broader lifespan process encompassing health, social participation, and access to services, and emerging evidence suggests distinct challenges in later adulthood. However, ageing in autism warrants more focused investigation beyond the scope of this umbrella synthesis.

## Discussion and conclusions

This umbrella review synthesised evidence on adverse outcomes experienced by autistic people across physical, mental, and social domains. It aimed to identify systematic associations, as identified in previous reviews and meta-analyses, to provide a comprehensive picture of autism-related outcomes. A key contribution of this review is the identification of cross-domain patterns of risk. Although each outcome is presented within organisational domains, the findings suggest that adverse outcomes rarely occur in isolation. Mental health difficulties, physical health conditions, and social disadvantage are likely to interact cumulatively across the lifespan. For example, sleep disturbance, gastrointestinal problems, and epilepsy may exacerbate emotional regulation difficulties; bullying and social exclusion may heighten anxiety and depression; and co-occurring mental health conditions may increase vulnerability to unemployment, victimisation, or reduced quality of life. We acknowledge how these adverse outcomes across mental, physical, and social domains may interact cumulatively and reinforce one another, while acknowledging that detailed causal modelling is beyond the scope of an umbrella review. These interacting risks highlight the limitations of siloed service responses and underscore the need for integrated, cross-sector models of care that recognise the compounding effects of mental, physical, and social challenges.

### Key findings and synthesis

This review provides a comprehensive synthesis of the most robust and consistent findings across mental health, physical health, and social domains, highlighting areas of significant challenge experienced by autistic people. The most reported adverse outcomes, drawing on a range of reviews published between 2013 and 2024, include mood disorders (with high prevalence rates ranging widely across studies due to methodological heterogeneity), suicide and self-harm (with strong evidence of increased risk compared to the general population), oral health issues (particularly increased plaque index and bruxism in children), feeding and gastrointestinal problems (elevated risk across age groups), sleep difficulties (objectively measured sleep disturbances are common), victimisation (including bullying and sexual violence, occurring at significantly higher rates than in non-autistic peers), and relationship challenges (e.g., fewer friends, increased loneliness). These findings underscore the broad and significant impact of challenges faced by autistic people across the lifespan on various aspects of life beyond diagnostic criteria.

These findings have implications for improving access to autism diagnosis and developing effective interventions and support for autistic people to help prevent these adverse outcomes. We now explore the main findings in greater depth and their relevance to clinical practice, with a view to improving support for autistic people. Many of the adverse outcomes identified are shaped not only by individual vulnerability but also by systemic and environmental factors, including variability in healthcare access, educational accommodation, and social support provision. The mechanisms through which these structural conditions disproportionately affect autistic individuals require further exploration.

### Mental health: implications and recommendations

Autistic people were at increased risk of co-occurring mental health conditions.

#### Anxiety and mood disorders

There is a consistent association between anxiety disorders (including OCD and social anxiety) and autism across the lifespan, and this association is also present for mood disorders such as bipolar disorder. However, findings for depression are less clear. The types of measurement tools used should be carefully considered in future research, particularly whether they are self- or parent-report and whether they are validated for autistic people (147). These results are a pertinent reminder that autism rarely occurs in isolation, and clinicians who make an autism diagnosis, especially in adults, need to remain curious about the potential for complex co-occurrence (148). In the absence of evidence-based post-diagnostic support for autistic adults and children, adequate treatment of related anxiety and mood problems in autistic people is imperative to mitigating these adverse outcomes (149).

#### Eating disorders

Autism was associated with eating disorders, with anorexia nervosa seemingly being the most common, followed by bulimia and binge eating disorder. More research is needed to pull apart overlapping characteristics and highlight potential diagnostic over- or under-shadowing between anorexia and autism (153). There is also a pressing need to develop comprehensive guidelines for treating eating disorders in the presence of possible autism (154). The potential link between autism and eating disorders may be mediated through a preference for routine and familiarity (e.g., same foods, mealtime routines), sensory processing differences (e.g., food taste, texture, smell) and difficulties with interoception (i.e., not being aware of hunger cues (155). Greater awareness of these processes in an autistic person’s support network could help overcome food-related challenges and may reduce the potential risk of developing an eating disorder. Supporting clinicians, parents and partners to create affirming environments that respect and validate autistic people’s experiences of body image and gender identity will also be of critical importance (156).

#### Psychotic and personality disorders

All reviews showed an association between psychotic disorders and autism. Individuals with schizophrenia have higher autistic characteristics, and autistic people are more likely to have Schizophrenia. Psychosis, psychotic experiences and paranoia are also more common in autistic people. These findings indicate the need for improved screening for psychosis, particularly in autistic adults who live more solitary lives (157). Less contact with friends and family can delay the identification of psychosis features, potentially allowing these to become well-established before the individual can access appropriate support (158). Enhancing social connectedness for autistic people, whether in person or virtually, may support earlier identification of emerging psychotic symptoms and reduce the impact of social isolation, which is a known risk factor for poorer mental health outcomes (159).

In contrast, evidence for an association between BPD and autism is inconsistent, with overlapping characteristics (e.g., interpersonal difficulties, emotional distress, vulnerability to trauma) complicating diagnosis (55). Misdiagnosis can occur when autistic traits are viewed through a BPD lens, particularly in autistic females who may adopt social camouflaging strategies to mask their difficulties. Clarifying the distinction and overlap between these conditions is essential for accurate assessment, reducing stigma, and ensuring appropriate, tailored support.

Moreover, while there is an association between PTSD and autism in children or adolescents, it is unclear whether these findings extend to adults (61). This is surprising given that PTSD prevalence generally increases with age due to cumulative trauma exposure (160), and autistic people are at an increased risk of experiencing potentially traumatic events and being significantly affected by them (161). Lower reported rates in adult autistic samples may therefore reflect differences in presentation or diagnostic challenges. Further research is needed to clarify this intersection, where trauma-informed and neurodiversity-affirming approaches to care are essential.

#### Substance misuse

There is some evidence that autistic people are more vulnerable to substance misuse, particularly in adulthood. However, this may not extend to autistic young people or females, where lower social connectedness and smaller peer groups might reduce exposure to peer pressure and act as protective factors against substance misuse (162). Conversely, lower social connectedness, social isolation and co-occurring mental health difficulties may increase the risk of alcohol dependency in some autistic people (163). More research is needed to understand developmental trajectories of substance misuse and how risk and protective factors vary across age, gender, and context.

#### Suicide and self-harm

The evidence suggests a significant association between autism and suicide or self-harm. Autistic people are more likely to self-harm (without suicidal intent) and are at an increased risk of all suicidality (ideation, behaviours and death) compared to the general population. The only review which implied otherwise was the first systematic review of suicidality in autism, at a time when evidence was limited (65). Further, a greater understanding of the complex interplay of suicide and self-harm risk factors is needed, primarily as other outcomes highlighted in this review, such as co-occurring mental health conditions, loneliness, and bullying victimisation, are known to heighten this risk in autistic people (164, 165). Currently, pilots of Autism Adapted Safety Plans and Dialectic Behaviour Therapy are the only suicide prevention interventions explicitly tailored for autistic people (166, 167). Autism-adapted suicide prevention approaches should incorporate structured emotional regulation interventions within both community and specialist mental health services. For example, skills-based emotion regulation programmes, such as adaptations of Dialectical Behaviour Therapy (168), should be formally integrated into care pathways for autistic people presenting with self-harm or suicidality, rather than delivered solely through generic provision alone. Embedding such interventions within routine service models may help reduce future suicide and self-harm risk.

### Physical health

Autistic people were at a higher risk of experience a range of physical health difficulties.

#### Oral health

Evidence for increased plaque index and bruxism (teeth grinding) in autistic children and adolescents was consistent, although there were mixed results for gingivitis, periodontal disease, and dental caries. Greater attention is required to oral health behaviours for autistic people, especially children. This may involve greater consideration of the type of toothbrush, as electric toothbrushes, which dentists recommend, are often too noisy. Additionally, there is a need for greater thought and education for parents about sensory factors that may influence the use of toothpaste or mouthwash, which are known to help address oral health problems (169). Technology can provide a valuable aid, such as smart apps, to support toothbrushing in autistic children (170), but further evaluation is still required.

#### Atopic dermatitis

An association between autism and atopic dermatitis (AD) was often demonstrated. AD is significantly more prevalent in autistic people compared to the general population, and autistic people are over-represented considerably in those diagnosed with AD. A greater understanding of the shared genetic and biological factors, especially in primary care, may help prevent parents of young autistic children and AD from being recommended topical solutions, as these may be unsuitable and distressing for autistic children or those with sensory needs (85).

#### Diabetes

Evidence of increased risk of diabetes in autistic people was mixed, with a wide range of prevalence estimates between studies. Higher quality studies that controlled for confounding variables often did not find a significant association between autism and diabetes, suggesting that factors such as age, obesity, psychotropic medication use, and co-occurring metabolic conditions may be stronger predictors of diabetes risk and account for the observed associations in some studies (88).

#### Asthma

Likewise, mixed evidence was found for increased prevalence of asthma in autistic children and adolescents, with some meta-analyses showing an association and others not. The reasons for this heterogeneity remain unclear, warranting further research to elucidate any potential association between asthma and autism. However, it may still be important for clinicians and parents to be aware that the most likely explanation for the association is genetic (171).

#### Epilepsy

On the other hand, evidence of an association between epilepsy and autism was found. There is increased prevalence of epilepsy in autistic people compared to the general population and those with ADHD, and autistic people are over-represented in those diagnosed with epilepsy. Increased age and co-occurring intellectual disability were risk factors for epilepsy in autistic people. Autism is often diagnosed later in children with epilepsy, which adds to the challenges faced by the autistic person and their families, who already live with the uncertainty of seizures, and must then navigate and make sense of an additional diagnosis (172).

#### Sleep

Robust evidence indicates an increased prevalence of sleep disorders and sleep problems in autistic people. For specific sleep difficulties, the strongest evidence comes from objective measures, including higher rates of sleep-disordered breathing, more frequent nocturnal awakenings, longer sleep-onset latency, and reduced total sleep time and sleep efficiency. There was also evidence that sleep problems are associated with several core autistic characteristics and subsequent behavioural problems in autistic children and adolescents. Given the secondary impact of sleep problems on families, there is a strong need to identify effective and parent-friendly interventions. The reduction or resolution of sleep problems has the potential for significant collateral benefits for the daytime functioning and well-being of autistic children and their families (173). Among autistic adults, Halstead (174) reported that 58% never had a visit with a healthcare professional regarding their sleep problem, despite 90% meeting the criteria for poor sleep quality. Therefore, greater awareness of sleep difficulties among healthcare professionals and better access to care are paramount.

#### Unhealthy weight/obesity

There was evidence of increased risk of obesity in autistic people, with risk increasing between 2 and 5 years old, whereas evidence for being underweight was mixed. Factors contributing to obesity risk among autistic children include the effects of psychotropic medication, lower levels of physical activity, and consumption of high-calorie foods and drinks (103). Greater awareness and support for autistic children and their families to promote exercise and make informed food choices would help lower this risk. Moreover, differences in eating behaviours, such as food selectivity or refusal due to sensory sensitivities, preference for structured mealtimes, and/or strong emotional responses from uncertainty around new foods, may also contribute to an unhealthy lower weight in some autistic children (105). Thus, additional dietary advice and support for families may be beneficial in meeting nutritional needs while respecting individual preferences.

#### Feeding problems/gastrointestinal problems

Reviews highlighted poor definitions of feeding problems and food sensitivity and a lack of high-quality studies on this topic in autistic people. Despite this, studies showed a high prevalence of these issues in autistic infants and young children, which were associated with an increased risk of nutritional deficiencies. Autistic people also had an increased risk of food allergies and intolerance, and were over-represented among those with these conditions. Additionally, there was consistent evidence of increased prevalence of gastrointestinal symptoms in autistic people, most commonly abdominal pain, constipation, diarrhoea, and broader functional bowel disturbances. Studies also suggested an association between some gastrointestinal diseases with autism, specifically eosinophilic gastrointestinal disease, irritable bowel disorder, ulcerative colitis and Crohn’s disease, but not celiac disease (175). These findings highlight the need for increased awareness and education of clinicians among gastrointestinal services to ensure appropriate, informed support for autistic people, as well as the importance of recognising and better understanding this link.

#### Vision and/or hearing impairment/loss

Evidence for increased prevalence of vision and/or hearing impairment or loss among autistic people was mixed, with a wide range of prevalence estimates across studies (e.g., 0%-87.8% for hearing impairment and 0%-15.3% for visual impairment). This likely reflects significant heterogeneity across study populations and methodologies among the included reviews, and further research is needed to examine the association between autism and vision and hearing impairments (176). Despite this, such associations, especially in children, highlight the importance of considering autism in assessments of those with a hearing or visual disability, to avoid missed or delayed diagnoses (177).

#### Mortality

High-quality evidence indicated at least twice the risk of mortality in autistic people compared to the general population. Risk factors for mortality included co-occurring intellectual disability (with higher rates of mortality and lower mean age of death compared to autistic people without intellectual disability), having epilepsy, and female sex. Increased mortality in autistic people likely reflects, in part, disparities in access to timely healthcare and the management of co-occurring conditions, such as epilepsy and mental health problems. Addressing this gap requires structured annual health checks, proactive monitoring of high-risk conditions, and improved coordination between primary care and specialist services. Embedding these measures within routine autism care pathways may help reduce preventable mortality and narrow observed health disparities (123).

#### Other physical health conditions

Additional reviews suggest various other physical health conditions which are experienced by autistic populations. For example, a higher prevalence of incontinence in autistic children compared to controls underscores the need for greater awareness of developmental and sensory differences that may affect toileting, and adequate assessment and treatment (178). Meta-analyses also showed a high prevalence of catatonia (10.4%) and an over-representation of autistic people in those with congenital heart disease (179). Moreover, the association between increased risk of a range of neurological conditions experienced by autistic people compared to the general population (e.g., macrocephaly, hydrocephalus, cerebral palsy, migraine/headache, congenital abnormalities of the nervous system) emphasises the need for integrated, multidisciplinary care that addresses both neurodevelopmental and neurological aspects of health.

Finally, evidence regarding an association between autism and allergy or autoimmune conditions remains mixed, and this pattern of inconsistency aligns with findings from a previous umbrella review. Collectively, this evidence calls for more nuanced and individualised approaches to understanding and supporting the diverse physical health needs of autistic people.

### Social and lifestyle: implications and recommendations

This final section explored the links between autism and many aspects of daily and social life.

Body Image and Identity.

While there is a significant relationship between negative body image and autistic traits, experiences are diverse and can also include positive aspects. Autistic people may experience body image differently, shaped by sensory differences and social pressures that lead to masking behaviours, rather than appearance concerns alone (125). However, most findings explore body image within clinical samples (e.g., eating disorders, weight management), limiting understanding across the broader autistic population. Additionally, evidence suggests that autistic people are more likely to identify as transgender or gender non-conforming (TGNC) (180). The mechanisms underlying this co-occurrence are not yet well understood; proposed hypotheses in the literature include differences in social cognition, identity development, and experiences of gender norms, but these remain areas of ongoing research. Given this intersectionality between autistic and TGNC individuals, and that both experience minority stress, the provision of affirming care is imperative to mitigate health disparities. There is a clear need to understand better how autism shapes experiences of body image and identity beyond clinical contexts (181).

#### Bullying, victimisation, and violence

All reviews showed that autistic people are at significantly higher risk of interpersonal harm, including peer bullying, sexual victimisation, and other forms of violence, compared to non-autistic peers. Generally, there is an emerging consensus that the relationship between autism and bullying is influenced by cumulative risk. There is a need to reduce the overall number of risks, rather than target specific risks, before we can reduce the extreme levels of bullying and victimisation that autistic people experience (182). It is also important to consider the role of internalising difficulties, such as anxiety, depression, and social withdrawal, which may increase vulnerability to bullying and victimisation, while also being worsened by these experiences, contributing to a reinforcing cycle of risk (183).

#### Gaming and internet use

Reviews suggest an association between autism and problematic gaming and internet use, though effect sizes were smaller and less consistent for problematic internet use. While the current evidence focuses on potential risks, there is limited exploration of the possible benefits of gaming for autistic people, such as providing predictable social contexts that support connection and a sense of belonging (184). Conversely, gaming in other areas of neurodiversity, such as ADHD (which frequently co-occurs with autism), is linked to providing reward sensitivity unavailable in other aspects of life (134). Nevertheless, core features of autism, such as hyperfocus and monotropic tendencies, may increase vulnerability to problematic use (135, 185). This supports a more nuanced, individualised understanding of how digital technologies are experienced.

#### Offending and criminality

Evidence across reviews indicates that autistic people as a group are not at increased risk of general offending or delinquent behaviour. A small number of studies have explored sexual offending specifically, with mixed and limited findings; where overrepresentation has been reported, authors emphasise contextual factors such as social misunderstanding, unmet support needs, or co-occurring conditions rather than autism itself. These findings should be interpreted with caution and do not indicate that autism is a causal risk factor for criminal behaviour. Payne et al. (186) qualitatively explored factors that may mean autistic people are more vulnerable to this type of crime. Autistic adults who had engaged in sexual offences self-reported difficulties with social skills, focused viewpoints, misunderstanding the seriousness of behaviours and a lack of appropriate support as the main reasons for offending. These findings highlight the need for early, tailored education, support, and safeguarding, rather than framing autism itself as a risk factor.

#### Quality of life

There is a consistent association between autism and a lower quality of life. However, measures used in studies have not been validated for use in autistic samples. They may fail to capture differences in aspects of life that autistic people value more/less than their non-autistic peers (187). Moreover, a mixed review suggested that the associations between various quality-of-life outcomes (e.g., employment, independence, social participation, relationships) and autism are inconsistent, likely due to methodological limitations, including the wide heterogeneity and variability of the measures used (18). Future research should prioritise the development of autistic-led or co-produced quality-of-life measurement tools that more accurately reflect the dimensions that matter most to autistic people.

#### Relationships, social connection, and sexual wellbeing

All reviews found an association between autism and relationship difficulties, social connection and sexual outcomes. Autistic children were reported to have fewer friends, lower quality relationships and less social participation status within traditional school environments. These difficulties appear to continue into adulthood, where autistic adults are also more likely to report loneliness. However, these outcomes are typically benchmarked against neurotypical social norms and may not capture what is personally meaningful or fulfilling for autistic people. Likewise, challenges faced by autistic people in initiating and navigating sexual relationships, having fewer opportunities for social connection, or a lack of accessible sexual health education can negatively affect understanding and expression of sexuality. Thus, tailored, affirming support that embraces neurodiversity is essential to promoting positive relational and sexual wellbeing for autistic people.

#### Other social and lifestyle outcomes

Further reviews found other social and lifestyle outcomes associated with autism. School absenteeism is more common in autistic children and adolescents than their non-autistic peers, partly attributable to co-occurring conditions and bullying as a risk factor (150). Educational disruptions may also contribute to problems later in life, such as incomplete education or the well-documented challenges that autistic adults face in accessing and remaining in meaningful employment (188). Likewise, a review highlighted unemployment itself as a significant adverse outcome experienced by autistic adults (39). Additionally, autistic people may encounter more challenges related to specific functional outcomes, such as driving (151), and a small number of reviews have also examined ageing in autism (152) compared to the general population. However, ageing represents a complex, lifelong biopsychosocial process rather than a discrete functional outcome, and warrants more focused and dedicated research beyond the scope of this umbrella review. Generally, current research often overlooks strengths, adaptations, and opportunities to improve outcomes for autistic people through inclusive, supportive approaches.

### Strengths and limitations

This is the first umbrella review to synthesise such a broad range of adverse outcomes experienced by autistic people, including physical, mental, social and lifestyle domains. As such, this review provides a valuable resource for clinicians and academics, presenting these outcomes together and demonstrating the full range of challenges and inequalities that autistic people may encounter throughout their lifespan and across various settings. With 134 reviews and over 1,300 studies included, this review has the unique advantage of consolidating years of research into a single publication.

The strengths of this review include its broad focus and thorough approach to selecting reviews for inclusion, which together provide a comprehensive analysis of the inequalities and challenges associated with autism across core domains that extend beyond the diagnostic criteria and their influence on daily life. Synthesising the evidence enabled us to identify various adverse outcomes faced by autistic people, providing a foundation for future research to explore underlying mechanisms and to inform more effective interventions and supports. It also provides insights to guide clinical and healthcare practices that promote better well-being for autistic people.

Additionally, the majority (85%) of the studies included in this review were deemed to be of good quality, with only two being rated as poor. The inclusion of high-quality reviews strengthens the validity of our findings. However, considerable heterogeneity in methodologies and statistical techniques across the reviews precluded meta-analysis or direct comparison of findings across publications. Because this umbrella review synthesises very broad outcome literature, domain classification required pragmatic organisational decisions. Domains were used as organisational categories rather than mutually exclusive constructs. Some constructs span multiple conceptual areas, and while transparent decision rules and reviewer consensus were used, a degree of thematic overlap is unavoidable in large-scale synthesis frameworks.

A key limitation of this umbrella review concerns variability and incomplete reporting of diagnostic ascertainment across included reviews. Although 66 reviews explicitly required DSM-, ICD-, or clinician-confirmed diagnoses, 5 permitted self-reported diagnosis alongside formal diagnosis, and 53 did not clearly specify diagnostic requirements. More broadly, reporting of diagnostic criteria was inconsistent, and many reviews did not clearly distinguish between formally diagnosed samples and self-identified autism. As this umbrella review synthesised 134 reviews comprising over 1,300 primary studies, it was not feasible to extract and verify diagnostic procedures at the level of individual studies. This variability in diagnostic definition and reporting likely contributes to heterogeneity in prevalence estimates and reported associations, and limits precision when interpreting the strength and comparability of findings across domains. Greater transparency and standardisation in reporting diagnostic inclusion criteria would strengthen future review-level syntheses. Given the breadth of the literature, a review of reviews was the most pragmatic approach to synthesise cross-domain evidence. This means that topics not synthesised through reviews may be omitted, potentially excluding important factors from consideration.

### Recommendations

The findings of this umbrella review underscore the urgent need for a holistic approach to the assessment and support for autistic people, acknowledging the widespread co-occurring conditions and impacts on various life domains. This review has implications for autistic people themselves, as well as for key stakeholders and professionals across healthcare, social, forensic, educational, and industrial sectors. The discussion above outlines several practice-level implications, but in relation to mental health conditions, our review highlights the need for clinicians to be mindful in assessing for co-occurring challenges related to substance use, addiction, suicide, eating disorders, mood and personality disorders. Given the consistent evidence of elevated anxiety, depression, and suicidality, adult and child autism diagnostic services should incorporate routine screening for common mental health conditions and routine suicide risk assessment at the point of diagnosis and during follow-up reviews.

Our review also indicates that clinicians should be aware of physical conditions such as gastrointestinal problems, oral hygiene, epilepsy, sleep and early mortality in relation to autism. Structured sleep and gastrointestinal assessment pathways should be integrated directly into clinical services. This is particularly pertinent, given that many autistic people report unmet needs and barriers to accessing healthcare (189, 190).

These findings have direct implications for policymakers, advocates, and service providers, underscoring the urgent need for systemic change and for holistic, tailored interventions. For example, the evidence of high rates of anxiety and depression among autistic adults necessitates the implementation of specific, autism-adapted screening protocols and evidence-based therapeutic interventions within adult mental health services. Furthermore, the high rates of victimisation and challenges in social connection highlight the imperative for anti-bullying initiatives and the development of accessible, neurodiversity-affirming social support programs.

Commissioners should ensure that autism-adapted psychological therapies are available within mainstream mental health services, rather than relying solely on generic provision. In light of the strong evidence of increased suicide risk, autism-adapted safety planning and structured crisis pathways should be embedded within community mental health services, and suicide prevention strategies at regional and national levels should explicitly recognise autistic people as a high-risk group (191). Furthermore, consistent associations with epilepsy, sleep disorders, gastrointestinal problems, and obesity highlight the need for integrated physical health monitoring protocols within autism care pathways, including structured annual health checks across primary and secondary care.

Beyond mental and physical healthcare, findings indicate the need for system-level responses across education, employment, and social care. Educational policies should include mandatory, enforced, anti-bullying strategies that explicitly address neurodiversity-related victimisation, alongside monitoring systems to enable early identification and intervention. Employment services should incorporate autism-informed workplace adjustment frameworks, including structured onboarding, sensory accommodations, and flexible communication supports. More broadly, the range of adverse outcomes identified across domains underscores the need for coordinated cross-sector approaches. Autism pathways should be embedded within integrated care and support models that recognise the cumulative interaction of mental, physical, and social barrier factors across the lifespan.

Future research should specifically focus on longitudinal studies exploring the causal pathways and the complex interplay between different adverse outcomes in autistic people, particularly examining the role of environmental factors and societal barriers in mediating these outcomes. Future studies should prioritise the use of validated measures, diverse samples, and clearer age stratification. We acknowledge that achieving these goals presents practical challenges, including recruitment barriers, resource constraints, and measurement limitations; however, gradual progress in these areas is essential to strengthening the robustness and applicability of the evidence base. Moreover, future research should explore the underrepresented areas of transgender and diverse gender identities and self-esteem. Intersectional factors such as race, gender, socioeconomic status, and co-occurring disability, particularly for underserved populations (192), are likely to shape exposure to adverse outcomes and access to support, but consistent extraction of these variables was not feasible within the scope of this umbrella review; this represents an important priority for future, focused, intersectionally informed syntheses.

This review, initiated to cover publications from inception, highlights that insights from earlier reviews, especially those pre-dating more refined diagnostic criteria (e.g., DSM-5 or ICD-11), may contribute to observed heterogeneity. Future syntheses could consider a more recent start date to align with current diagnostic understanding, provided a strong justification is offered for this temporal scope.

## Data Availability

The original contributions presented in the study are included in the article/**Supplementary Material**. Further inquiries can be directed to the corresponding author.

## Footnotes

1 We have opted to use identity first language (autistic people), as previous research has shown that this is the most highly endorsed preference by the autistic community. However, we acknowledge that a wide range of language is used to describe autism which will continue to develop and change over time.
