# Supplementary material for: Adverse outcomes for autistic people: an umbrella review of mental health, physical health, social and lifestyle domains
Source: Front Psychiatry. 2026 May 11;17:1702822. doi: 10.3389/fpsyt.2026.1702822 (PMC13200339; doi:10.3389/fpsyt.2026.1702822)
Supplement: Supplementary file 1 [file Table1.docx]

# Table 1 - Included studies

| Mental health | | | | | | | | | | | | | | | | | | |
| --- | --- | --- | --- | --- | --- | --- | --- | --- | --- | --- | --- | --- | --- | --- | --- | --- | --- | --- |
| First author, year | Title of review | | Year of studies (range) | | Topic comparison | | | Population of interest | | Number of studies included | | Type of review | | Synopsis of findings | | Quality assessment score | Quality assessment rating | |
| Multiple condition reviews | | | | | | | | | | | | | | | | | | |
| Barlattani, 2023 | Autism spectrum disorders and psychiatric comorbidities: a narrative review | | NR | | Review of psychological disorders | | | Both (age range NR) | |  | | Narrative review | | ADHD has the highest prevalence among psychiatric comorbidities in autism, followed by anxiety disorders. The clinical presentation of all the disorders considered is often overlapping | | 1 | Good* | |
| Bougeard, 2021 | Prevalence of Autism Spectrum Disorder and Co-morbidities in Children and Adolescents: A Systematic Literature Review | | 2012-2017 | | Comorbidities | | | Children and adolescents (age range 0-24 years) | | 13 | | Systematic review | | The review highlighted wide prevalence ranges for each co-morbidity: 0.0–86% for ADHD (17/33 studies), 0.0–82.2% for anxiety (13/33 studies), 0.0– 38.6% for depressive disorders (12/33 studies), 2.8–43.75% for epilepsy/seizures (12/33 studies), 0.0–49.0% for GI syndromes (7/33 studies), 0.0–87.8% for hearing impairment (3/33 studies), 0.0–91.7% for ID, 6.4–72.5% for sleep disorders (5/33 studies) and 0.0–15.3% for visual impairment (3/33 studies). | | 0.7 | Good | |
| Hossain, 2020 | Prevalence of comorbid psychiatric disorders among people with autism spectrum disorder: An umbrella review of systematic reviews and meta-analyses | | 2006-2019 | | Umbrella of psychiatric disorders | | | Both (mean age range 2-40.3 years) | | 26 | | Umbrella review | | The synthesized findings reveal a high burden of comorbid psychiatric disorders among autistic people, including anxiety disorders, depressive disorders, bipolar and mood disorders, schizophrenia spectrum, suicidal behavior disorders, attention-deficit/hyperactivity disorder, disruptive, impulse-control and conduct disorders amongst diverse age groups, with a majority in younger participants. | | 0.82 | Good | |
| Howlin, 2017 | Autism spectrum disorder: Outcomes in adulthood. | | 2015-2016 | | Adult outcomes | | | Adults | | 43 | | Literature review | | Overall outcomes in terms of jobs, relationships, independent living and mental health are poorer for autistic adults than for same age peers | | 1 | Good* | |
| Lai, 2019 | Prevalence of co-occurring mental health diagnoses in the autism population: a systematic review and meta-analysis | | 2006-2019 | | Review of mental health diagnosis | | | Both (age range 2-95 years) | | 100 | | Systematic review and meta-analysis | | 96 studies were meta-analysed. Overall pooled estimates were for ADHD 28% (95%CI 25-32%), anxiety disorders 20% (17-23%), sleep-wake disorders 13% (9-17%), disruptive/impulse-control/conduct disorders 12% (10-15%), depressive disorders 11% (9-13%), obsessive-compulsive disorder 9% (7-10%), bipolar disorders 5% (3-6%) and schizophrenia spectrum disorders 4% (3-5%). | | 0.95 | Good | |
| Lugo-Marin, 2019 | Prevalence of psychiatric disorders in adults with autism spectrum disorder: A systematic review and meta-analysis | | 2006-2016 | | Review of psychiatric disorders | | | Both (age range 14-84 years) | | 47 | | Systematic review and meta-analysis | | Results showed that attention deficit and hyperactivity disorder is the most prevalent psychiatric disorder in autistic adults. Mood and anxiety disorders are also very frequent among this population. The lowest comorbidity prevalence rates of all diagnostic categories are the ones related to substance use and eating disorders. | | 1 | Good | |
| Matson, 2013 | Comorbidity and autism: Trends, topics and future directions | | NR | | Corbidities | | | Both (age range NR) | | 449 | | Literature review | | High rates of comorbidity between personality and anxiety disorder were found. Similarly, ADHD symptoms have been commonly reported among persons with bipolar disorder (Masi et al., 2006, Wildes and Marcus, 2013). A host of other conditions have also been reported as co-occurring. | | 0.67 | Moderate* | |
| Micai, 2023 | Prevalence of co-occurring conditions in children and adults with autism spectrum disorder: A systematic review and meta-analysis | | 1984-2021 | | Co-occuring conditions | | | Both (mean age range 0.9-67 years) | | 340 | | Systematic review and meta-analysis | | Among the mental health/psychiatric co-occuring conditions (ccs), the most frequently reported ccs, with their point pooled prevalence estimates, were developmental coordination disorder, sleep-wake problem, ADHD, anxiety disorder, ID (point and lifetime prevalence pooled together), feeding and eating disorder, disruptive behavior, somatic symptom and related disorders, and sleepwake disorder. Among the neurological and medical CCs, the most frequent overall point prevalence was the following: motor problem, GI  problem, overweight/obesity, elimination disorder, organic nutrition disorder, and GI disorder. Prevalence of ADHD, sleep-wake problem, somatic symptom and related disorders, and celiac disease were higher  in children/adolescents compared to adults. While, the prevalence of motor problem, GI disorder, depressive disorder, epilepsy, hearing disorder, and neurocutaneous disorder were higher in adults compared to  children/adolescents. | | 0.91 | Good | |
| Mutluer, 2022 | Population-Based Psychiatric Comorbidity in Children and Adolescents With Autism Spectrum Disorder: A Meta-Analysis | | 2005-2019 | | Psychiatric comorbidities review | | | Both (age range 14-84 years) | | 29 | | Meta-analysis | | The main findings show prevalence estimates of 22.9% (95% CI: 17.7- 29.2) for intellectual disability; 26.2% (22-31) for attention-deficit hyperactivity disorder; 11.1% (8.6-14.1) for anxiety disorders; 19.7% (11.9-30.7) for sleep disorders; 7% (5.2- 9.3) for disruptive disorders; 2% (1.3- 3.1) for bipolar disorders; 2.7% (1.8- 4.2) for depression; 1.8% (0.4–8.7) for obsessive-compulsive disorder; and 0.6% (0.3–1.1) for psychosis. Psychiatric comorbidity in population-based studies is lower than in clinical and referred samples. | | 0.73 | Good | |
| Rosen, 2018 | Co-occurring psychiatric conditions in autism spectrum disorder | | NR | | Co-occuring psychiatric conditions | | | Both (age range NR) | | NR | | Literature review | | Co-occurring disorder research in austism has been complicated by several issues, including diagnostic overshadowing of autism symptoms over psychiatric symptoms, and vice versa, atypical or ambiguous presentations of co-occurring symptoms, and emotional dysregulation inherent to AUTISMthat may hinder accurate assessment or effective treatment. However, it is clear that many autistic youth are negatively affected by the presence of one or more ‘true’ co-occurring psychiatric conditions. | | 0.83 | Good* | |
| Anxiety and mood disorders | | | | | | | | | | | | | | | | | | |
| González, 2024 | The childbearing and mental health experiences of autistic mothers: a systematic review | | 2013-2022 | | Childbearing and mental health | | | NR | | 15 | | Systematic review | | Autistic mothers is more likely to experience parenting difficulties, intense sensory experiences and mental health problems. However, two studies showed that these mental health problems were not significant when compared to non-autistic mothers and that autistic women were able to still maintain marital satisfaction despite the difficulties around pregnancy and birth. | | 1.00 | Good | |
| Hollocks, 2021 | Anxiety and depression in adults with autism spectrum disorder: a systematic review and meta-analysis | | 2004-2017 | | Anxiety and depression | | | Both (age range 16-79 years) | | 35 | | Systematic review and meta-analysis | | Autistic adults experience high rates of comorbid anxiety and depression. Both anxiety and depression are prominent and common in adults with a diagnosis of autism. The exact prevalence is difficult to estimate precisely, given high levels of heterogeneity between studies, but our results suggest rates significantly higher than one would expect. | | 0.77 | Good* | |
| Kilanko, 2022 | The Correlation Between Autistic Childhood Disorders and the Development of Anxiety and Depression in Adults: A Systematic Review | | 2009-2020 | | Anxiety and depression | | | Adults (age range NR) | | 5 | | Systematic review | | The study shows psychiatric disorders like anxiety and depression could be related to autism spectrum disorder | | 0.50 | Moderate | |
| Menezes, 2018 | Depression in Youth with Autism Spectrum Disorders: a Systematic Review of Studies Published Between 2012 and 2016 | | 2012-2016 | | Depression | | | Children and adolescents (age range 1-20 years) | | 43 | | Systematic review | | The results of the review indicate that depression is more common in autistic youth than in typically developing youth and is associated with a multitude of other medical and psychiatric conditions | | 0.70 | Good | |
| Schwartzman, 2022 | Depression and Employment Outcomes in Autistic Adults: A Systematic Review | | 2011-2021 | | Depression and unemployment | | | Adults (mean age range 20.4–61.5 years) | | 21 | | Systematic review | | Clinically-significant depressive symptoms were frequently reported by autistic adults (within-sample mean of 44% autistic adults with elevated scores), while active employment was less frequently reported (26–55% of adults employed in some capacity). Severe depression and/or suicidality were more common in autistic adults than non-autistic adults, while full-time employment was less common in autistic adults. Of the 10 studies that empirically tested associations between depression and employment, findings suggest that employment does not predict depression and/or suicidality. However, depression may interfere with employment retention and quality of life for some autistic adults. | | 0.60 | Moderate | |
| Spain, 2018 | Social anxiety in autism spectrum disorder: A systematic review | | 2004-2016 | | Social anxiety (SA) | | | Both (age range 5-57 years) | | 25 | | Systematic review | | Social anxiety (SA), in autistic individuals , was associated with poorer social skills and functioning, and reduced social motivation. There were associations between self-report SA and autism measures, but a trend towards non-significant relationships between parent-ratings of these symptoms. Tentative evidence indicated that SA symptoms were not associated with restricted, repetitive behaviours or sensory sensitivities. | | 0.80 | Good | |
| Syriopoulou-Delli, 2022 | A systematic review of dysfunctional thoughts, feelings and phobias of children and adolescents with autism. Solutions and therapeutic methods | | 2003-2020 | | Anxiety and depression and ocd | | | Children and adolescents (age range 5-18 years) | | 15 | | Systematic review | | Autistic children and adolescents show various symptoms of psychological disorders such as Anxiety Disorders, Depression and Obsessive-Compulsive Disorder. | | 0.65 | Moderate | |
| Tracy, 2021 | A systematic review of the rates of depression in autistic children and adolescents without intellectual disability | | 1998-2016 | | Depression | | | Both (age range 4.5-51 years) | | 19 | | Systematic review | | Rates of depression in autistic children and adolescents vary considerably across studies (from 0% to 83.3%) and do not show particular pattern in relation to methodoly or age. | | 0.95 | Good | |
| Van Steensel, 2011 | Anxiety disorders in children and adolescents with autistic spectrum disorders: a meta-analysis | | 1979-2010 | | Anxiety disorders | | | Children and adolescents (mean age range 4.2-16.3 years) | | 31 | | Systematic review and meta-analysis | | The results reveal substantial comorbidity for anxiety in children and adolescents with nearly 40 percent of autistic young people had at least one comorbid DSM-IV anxiety disorder, the most frequent being specific phobia (29.8%) followed by OCD (17.4%) and social anxiety disorder (16.6%). | | 0.73 | Good | |
| Van Steensel, 2017 | Anxiety levels in children with autism spectrum disorder: A meta-analysis | | 1990-2016 | | Anxiety | | | Children and adolescents (mean age range 3.54-18.54 years) | | 83 | | Meta-analysis | | (1) anxiety levels of autistic youth are much higher compared to typically developing children (large effect size difference); (2) anxiety levels of autistic children seem elevated compared to clinically referred children in general (small effect size difference); (3) the type of comparison group seems to matter in the direction that anxiety levels in autistic youth were found to be higher compared to youth with externalizing or developmental problems, but compared to children with internalizing problems results were inconsistent for the fixed and random model. | | 0.73 | Good | |
| Vannucchi, 2014 | Bipolar disorder in adults with Asperger's Syndrome: a systematic review | | 1991-2011 | | Bipolar | | | Both (age range 5-60 years) | | 7 | | Systematic review | | Bipolar disorder (BD) prevalence in autistic adults ranges from 6% to 21.4% of the cases. The literature also strongly supports that autism may be associated with a family history for mood disorders and that the most frequent association is reported with BD in family members of autistic patients. | | 0.90 | Good | |
| Wigham, 2017 | A systematic review of the rates of depression in children and adults with high-functioning autism spectrum disorder | | 2010-2015 | | Depression | | | Both (mean age range 8.3-41.57 years) | | 19 | | Systematic reivew | | Rates of depression in high-functioning autistic people varied widely across studies, from 1% to 47.1%. Most rates were higher than the general-population prevalence rates of 2.5% to 10.7%. However, confidence in the validity of comparing these rates of MDD in high functioning autistic people and general-population prevalence rates is compromised by a number of factors related to methodological aspects of the studies. | | 0.90 | Good | |
| Eating disorders | | | | | | | | | | | | | | | | | | |
| Boltri, 2021 | Anorexia Nervosa and Autism Spectrum Disorder: A Systematic Review | | 2017-2021 | | Anorexia | | | Both (mean 9.91 -42 years) | | 13 | | Systematic review | | Analysed studies suggest that while the comorbidity seems to exacerbate the severity of the condition, autistic traits appear to be rather stable over time and not related to body weight. Thus, future longitudinal studies and gender- specific assessment tools can help clarify the relationship between the two disorders, examine the onset of autism symptoms and develop structured guidelines for treatment. | | 0.70 | Good | |
| Huke, 2013 | Autism spectrum disorders in eating disorder populations: A systematic review | | 1992-2011 | | Eating disorder | | | Both (mean age range 16-24 years; age range 18-56 years) | | 8 | | Systematic review | | Prevalence rates of autism in an eating disorder population have been found to be significantly higher than those of healthy control participants. However, as a large number of results come from the same sample, more varied future samples are required to limit the possibility of bias. | | 1 | Good | |
| Nickel, 2019 | Systematic Review: Overlap Between Eating, Autism Spectrum, and Attention-Deficit/Hyperactivity Disorder | | NR | | Eating disorders | | | Both (mean age range 11.6-27.4 years) | | 17 | | Systematic review | | On average, 4.7% of patients with certain eating disorder (ED) diagnoses (anorexia nervosa, bulimia nervosa, and binge eating disorder) received an autism diagnosis. Studies on the prevalence rates of ED in autism and vice versa are heterogeneous, but they indicate frequent association. | | 0.60 | Moderate | |
| Westwood, 2017 | Autism Spectrum Disorder in Anorexia Nervosa: An Updated Literature Review | | 2012-2017 | | Anorexia Nervosa (AN) | | | Both (mean age range 11.6-26.4 years) | | 8 | | Literature review | | The wide range of diagnostic tools, methodologies and populations studied make it difficult to determine the prevalence of autism in AN. Despite this, studies consistently report over-representation of symptoms of autism in AN | | 0.75 | Good* | |
| Psychotic and personality disorders | | | | | | | | | | | | | | | | | | |
| Curnow, 2023 | Mental health in autistic adults: A rapid review of prevalence of psychiatric disorders and umbrella review of the effectiveness of interventions within a neurodiversity informed perspective | | NR | | Psychiatric disorders | | | Both (3-79 years age range) | | 52 | | Rapid review | | There is limited understanding of mental ill-health and how this can impact quality of life for autistic people despite evidence indicating increased prevalence. There is a need for diagnostic tools and outcome measures to be validated for use with this population. Future research should fully include autistic people at every stage and focus on priorities identified by the autistic population. | | 1 | Good* | |
| De Crescenzo, 2019 | Autistic symptoms in schizophrenia spectrum disorders: A systematic review and meta-analysis | | 2001-2017 | | Schizophrenia (SSDS) | | | Both (mean 6.7 - 42.2 years) | | 13 | | Systematic review and meta-analysis | | Individuals with SSDS have higher autistic symptoms than healthy controls and lower autistic symptoms than autistic individuals. | | 0.77 | Good | |
| Dell’Osso, 2023 | Comorbidity and Overlaps between Autism Spectrum and Borderline Personality Disorder: State of the Art | | 2006-2021 | | borderline personality disorder (BPD) | | | adults (mean age range 20.3-39.43) (NR in all studies) | | 13 | | Literature review | | Despite some controversial results and lack of homogeneity in the methods used for the diagnostic assessment, the reviewed literature highlighted how subjects with BPD reported higher scores on tests evaluating the presence of AT compared to a non-clinical population and hypothesized the presence of unrecognized autism in some BPD patients or vice versa, while also describing a shared vulnerability towards traumatic events, and a greater risk of suicidality in BPD subjects with high autistic traits. | | 0.75 | Good* | |
| Kincaid, 2017 | What is the prevalence of autism spectrum disorder and ASD traits in psychosis? A systematic review | | 2003-2015 | | Psychosis | | | Both (age range 13-93 years) | | 7 | | Systematic review | | This review demonstrates elevated prevalence rates of autism at the diagnostic level and at the trait level in psychotic populations compared to the general population. The co-occurrence of psychosis and alts is evidently more frequent than autism at the diagnostic level. | | 0.95 | Good | |
| Kiyono, 2020 | The Prevalence of Psychotic Experiences in Autism Spectrum Disorder and Autistic Traits: A Systematic Review and Meta-analysis | | 1998-2017 | | Psychotic experiences | | | Both (mean age range 8 months-34.98 years) | | 17 | | Systematic review and meta-analysis | | The pooled prevalence of psychotic experiences (PES) in autism was 24% (95% confidence interval [CI] 14%–34%). However, subanalyses found that prevalence varied between PE subtypes (hallucinations, 6% [95% CI 1%–11%] and delusions, 45% [95% CI 0%–99%]). Pooled results showed that pes and autistic traits had a weak to medium correlation (r = .34 [95% CI 0.27–0.41]). Based on our meta-analysis, PES seem to be more prevalent in autistic individuals or with autistic traits than in the general population, but this finding may vary according to the PE subtype. | | 0.91 | Good | |
| May, 2021 | Overlap of autism spectrum disorder and borderline personality disorder: A systematic review and meta-analysis | | 1998-2020 | | borderline personality disorder | | | both (mean age range 11-39.36 years) | | 19 | | Systematic review and meta-analysis | | The pooled prevalence of BPD in autism was 4% [95% CI 0%–9%] and of autism in BPD, 3% [95% CI 1%–8%]. There were inconsistent findings across clinical areas. The prevalence of a dual diagnosis of BPD in autism cohorts and of autism in BPD cohorts was within population prevalence estimates of each disorder. Based on this data we were not able to assess whether there is misdiagnosis of one in favor of the other. | | 0.77 | Good | |
| Rumball, 2019 | A systematic review of the assessment and treatment of posttraumatic stress disorder in individuals with autism spectrum disorders | | 1993-2017 | | PTSD | | | both (mean age range 6-30.5 years) | | 24 | | Systematic review | | PTSD in children and adolescents was found to co-occur at a similar or greater rate compared to general population estimates, although current estimates come predominantly from treatment-seeking samples. Preliminary findings from case reports suggest traditional assessments and treatments for PTSD can be effective, although there is a shortage of well-controlled research. | | 0.9 | Good | |
| Spain, 2016 | Conceptualising paranoia in ASD: A systematic review and development of a theoretical framework | | 2001-2015 | | Paranoia | | | Both (mean age range 16-42 years) | | 7 | | Systematic review | | Autistic individuals were consistently found to have higher levels of paranoia compared to non-clinical controls, and lower levels than individuals with current psychotic experiences manifesting in the context of schizophrenia. Furthermore, the initial evidence indicates that paranoia in autism may be linked with theory of mind performance, negative affect, and jumping to conclusions, but not to attributional style. | | 0.75 | Good | |
| Varcin, 2022 | Occurrence of psychosis and bipolar disorder in adults with autism: A systematic review and meta-analysis | | 2003-2021 | | Psychosis and bipolar | | | Both (age range 11-96 years) | | 53 | | Systematic review and meta-analysis | | The pooled prevalence for the co-occurrence of psychosis in autistic adults was 9.4 % (N =63,657, 95 %CI =7.52, 11.72). The pooled prevalence for the co-occurrence of bipolar disorders in autistic adults was 7.5 % (N =31,739, 95 %CI =5.79, 9.53). | | 1 | Good | |
| Zheng, 2018 | Association between schizophrenia and autism spectrum disorder: A systematic review and meta-analysis | | 2004-2016 | | Schizophrenia | | | Both (mean age range 4.9-30.6 years) | | 17 | | Systematic review and meta-analysis | | The prevalence of schizophrenia was significantly higher in autistic individuals than in controls (odds ratio = 3.55, 95% confidence interval: 2.08–6.05, P < .001). The prevalence of autism in individuals with schizophrenia ranged from 3.4 to 52%. T | | 0.77 | Good | |
| Substance misuse | | | | | | | | | | | | | | | | | | |
| Arnevik, 2016 | Autism Spectrum Disorder and Co-occurring Substance Use Disorder - A Systematic Review | | 2006-2015 | | substance abuse (SUD) | | | both (age N/R) | | 18 | | Systematic review | | In most of the treatment settings studied, there were relatively few patients with co-occurring autism and SUD, but due to differences in study samples it was difficult to establish a general prevalence rate. The one consistent finding was the lack of focused treatment studies. There is clearly a need for research on interventions that take account of the special needs of this patient group. | | 0.70 | Good | |
| Haasbroek, 2022 | A Systematic Literature Review on the Relationship Between Autism Spectrum Disorder and Substance Use Among Adults and Adolescents | | 2009-2019 | | Substance use | | | Both (age range 13-65 years) | | 26 | | Systematic review | | This systematic literature review concludes that there is indeed increased comorbidity, vulnerability or risk factor for those within the autistic population to develop some type of SUD, but this finding was not true for adolescents and younger people. This may be due to the age of onset of substance use within this population, especially as this population demonstrates various developmental delays. | | 0.55 | Moderate | |
| Ressel, 2020 | Systematic review of risk and protective factors associated with substance use and abuse in individuals with autism spectrum disorders | | 2009-2019 | | Substance abuse | | | Both (age range 6-71 years) | | 26 | | Systematic review | | Prevalence rates of substance abuse among autistic samples ranged from 1.3% to 36%, but due to variability in sample characteristics and diagnostic measures, a general prevalence rate could not be established. Risk and protective factors, recognized in the general population, such as familial substance abuse and comorbid externalizing disorders, and factors, which may be more likely to occur in autistic individuals compared to the general population, such as few social resources (i.e. Sense of social belonging, breadth of social support networks, and level of social capital) and low sensation-seeking, were identified. | | 0.85 | Good | |
| Suicide and self-harm | | | | | | | | | | | | | | | | | | |
| Blanchard, 2021 | Risk of Self-harm in Children and Adults With Autism Spectrum Disorder: A Systematic Review and Meta-analysis | | 1999-2020 | | Suicide | | | Both (0-100 years) | | 33 | | Systematic review and meta-analysis | | This systematic review with meta-analysis found that autism was associated with a substantially increased risk of self-injurious behaviors and suicidality. This finding was consistent in pediatric and adult populations across geographic regions and in study designs, methods, and settings. Further research is needed to examine the role of primary care screenings, preventive mental health services, and lethal means counseling in reducing self-harm among autistic people. | | 0.86 | Good | |
| Figueiredo, 2023 | Self-injurious behaviors in children and adolescents with autism spectrum disorder without intellectual disability | | 2010-2020 | | Self injury behaviour (SIB) | | | Both (age range 2-21 years) | | 14 | | Systematic review | | The prevalence rates of SIB in the sample ranged between 10.1–70.5%. The higher rates of prevalence are from inpatient samples or day-hospital services, as expected. | | 0.50 | Moderate | |
| Hannon, 2013 | Suicidal behaviour in adolescents and young adults with ASD: findings from a systematic review | | 2005-2010 | | Suicide behaviour | | | Children and adolescents (a mean age range of 6-20 years) | | 4 | | Systematic review | | In light of the evidence reviewed thus far suicidal behaviours appear to be a clinically important problem in autistic young people. The rate of suicidal behaviours in the studies reviewed appears to be similar to the rate among the typically developing population. | | 0.85 | Good | |
| Hedley, 2018 | Systematic Review of Suicide in Autism Spectrum Disorder: Current Trends and Implications | | 2013-2017 | | Suicide | | | Both (age range 7-146 years) | | 13 | | Systematic review | | There is increasingly strong evidence that suicide is a critical issue facing autistic people—recent studies report increased suicidal thoughts, behaviour, and premature death by suicide compared to both general and clinical populations. | | 0.70 | Good | |
| Huntjens, 2023 | The prevalence of suicidal behaviour in autism spectrum disorder: A meta-analysis | | 1990-2022 | | Suicide attempts and ideation | | | Both (age range 5-65 years) | | 52 | | Systematic review and meta-analysis | | Pooled estimates for 12 months (SI: 25.4%, SA:14.1%) and the lifetime (SI: 37.2%, SA: 15.3%) were consistently higher than the general population’s estimates of 2.0% for SI and 0.3% for SA over 12 months and 9% for SI and 3% for SA over a lifetime. | | 1.00 | Good | |
| Newell, 2023 | A systematic review and meta-analysis of suicidality in autistic and possibly autistic people without co-occurring intellectual disability | | 2000-2021 | | Suicide | | | Both (age range 7 - 75 years) | | 36 | | Systematic review and meta-analysis | | Pooled prevalence of suicidal ideation was 34.2% (95% CI 27.9–40.5), suicide plans 21.9% (13.4–30.4), and suicidal attempts and behaviours 24.3% (18.9–29.6). High levels of heterogeneity (I2>75) were observed in all three analyses. | | 0.91 | Good | |
| O'Halloran, 2022 | Suicidality in autistic youth: A systematic review and meta-analysis | | 2000-2021 | | Suicide | | | Children and adolescents (mean age range 7.7-21.5 years) | | 47 | | Systematic review and meta-analysis | | 29 of 47 were included in meta-analyses and 37 were narratively synthesized. The pooled prevalence of suicidal ideation was 25.2% (95% CI 18.2–33.8; i.e., one in four), suicide attempts 8.3% (3.6–18.2), and suicide deaths 0.2% (0.05–0.52). Estimates in self-reports were higher than in parent-reports. Age was a significant but inconsistent moderator on suicide outcomes, but substantial heterogeneity remained. Adverse childhood experiences were strong risk factors for suicidality, while resilience was protective. | | 0.95 | Good | |
| Oliphant, 2020 | What is the Prevalence of Self-harming and Suicidal Behaviour in Under 18s with ASD, With or Without an Intellectual Disability? | | 1995-2017 | | Suicide and self-harm | | | Children and adolescents (age range 5-20 years) | | 9 | | Systematic review | | The reported prevalence rates of self-harm and suicidal behaviour suggest that rates may be elevated in autism compared to the general population. This would be in keeping with fndings of adult studies and in contrast to conclusions of a previous systematic review. | | 0.90 | Good | |
| Segers, 2014 | What do we know about suicidality in autism spectrum disorders? A systematic review | | 1997-2013 | | Suicide | | | Both (mean age range 6.6-42.7 years) | | 13 | | Systematic review | | Suicidality was present in 10.9–50% of the autism samples identified in the systematic review. Further, several large-scale studies found that autistic individuals comprised 7.3–15% of suicidal populations, a substantial subgroup. Risk factors were identified and included peer victimization, behavioral problems, being Black or Hispanic, being male, lower socioeconomic status, and lower level of education. | | 0.60 | Moderate | |
| Steenfeldt-Kristensen, 2020 | The Prevalence of Self-injurious Behaviour in Autism: A Meta-analytic Study | | 1976-2018 | | Self-injury | | | NR | | 37 | | Systematic review and meta-analysis | | The overall pooled prevalence estimate for self-injury in autism is 42%, which is significantly higher than prevalence estimates for self-harm in the typically developing population. For example, the prevalence of self-harm in typically developing children and adolescents is approximately 8% and 5.9% in adults. The difference between these prevalence rates are striking and suggests that those autistic are a particularly high risk group for self-injury. | | 0.77 | Good | |
| Zahid, 2017 | Suicidality in autistic spectrum disorders: A systematic review | | NR | | Suicide | | | Both (age range NR) | | 12 | | Systematic reivew | | Prevalence of suicide attempts varied between 7% and 47%, while suicidal ideation was reported in up to 72% of cases. Being male and having a history of self-harm and depression were cited as significant risk factors. | | 0.61 | Moderate | |
| Physical Health | | | | | | | | | | | | | | | | | | |
| Multiple factors reviews | | | | | | | | | | | | | | | | | | |
| Bougeard, 2021 | | Prevalence of Autism Spectrum Disorder and Co-morbidities in Children and Adolescents: A Systematic Literature Review | | 2012-2017 | | comorbidities | children and adolescents (age range 0-24 years) | | 13 | | Systematic review | | The review highlighted wide prevalence ranges for each co-morbidity: 0.0–86% for ADHD (17/33 studies), 0.0–82.2% for anxiety (13/33 studies), 0.0– 38.6% for depressive disorders (12/33 studies), 2.8–43.75% for epilepsy/seizures (12/33 studies), 0.0–49.0% for GI syndromes (7/33 studies), 0.0–87.8% for hearing impairment (3/33 studies), 0.0–91.7% for ID, 6.4–72.5% for sleep disorders (5/33 studies) and 0.0–15.3% for visual impairment (3/33 studies). | | 0.70 | | | Good |
| Howlin, 2017 | | Autism spectrum disorder: Outcomes in adulthood. | | 2015-2016 | | adult outcomes | adults | | 43 | | Literature review | | Overall outcomes in terms of jobs, relationship, independent living and mental health are poorer for autistic adults that for same age peers | | 1* | | | Good* |
| Matson, 2013 | | Comorbidity and autism: Trends, topics and future directions | | NR | | corbidities | both (age range NR) | | 449 | | Literature review | | High rates of comorbidity between personality and anxiety disorder were found. Similarly, ADHD symptoms have been commonly reported among persons with bipolar disorder. A host of other conditions have also been reported as co-occurring. | | 0.67* | | | Moderate* |
| Micai, 2023 | | Prevalence of co-occurring conditions in children and adults with autism spectrum disorder: A systematic review and meta-analysis | | 1984-2021 | | co-occuring conditions | both (mean age range 0.9-67 years) | | 340 | | Systematic review and meta-analysis | | Among the mental health/psychiatric co-occuring conditions (CCs), the most frequently reported CCs, with  their point pooled prevalence estimates, were developmental coordination disorder, sleep-wake problem, ADHD, anxiety disorder, ID (point and lifetime prevalence pooled together), feeding and eating disorder,  disruptive behavior, somatic symptom and related disorders, and sleep-wake disorder. | | 0.91 | | | Good |
| Muskens, 2017 | | Medical comorbidities in children and adolescents with autism spectrum disorders and attention deficit hyperactivity disorders: a systematic review | | 2008-2015 | | medical comorbidities | both (age range 1-65 years) | | 14 | | Systematic review | | The main finding of this systematic review is that medical disorders in autistic children appear to be widespread, e.g., can manifest across different medical areas, such as immunology, neurology and gastroenterology. | | 0.70 | | | Good |
| Rydzewska, 2021 | | Umbrella systematic review of systematic reviews and meta-analyses on comorbid physical conditions in people with autism spectrum disorder | | 2008-2019 | | Umbrella review of physical conditions | both (age range 0-66 years) | | 24 | | Umbrella review | | Comorbid physical conditions are common, and some are more prevalent than in the general population: sleep problems, epilepsy, sensory impairments, atopy, autoimmune disorders and obesity. Asthma is not. However, there are substantial gaps in the evidence base. Fewer studies have been undertaken on other conditions and some findings are inconsistent. | | 0.85 | | | Good |
| Oral health | | | | | | | | | | | | | | | | | | |
| AlOtaibi, 2021 | | A systematic review of population-based gingival health studies among children and adolescents with autism spectrum disorder | | 2014-2017 | | Teeth, gingival index and plaque index | children (2-16 years) | | 5 | | systematic review | | Autistic children and adolescents have poorer oral hygiene and higher rates of gingival disease than non-autistic individuals. | | 0.64 | | | Moderate |
| Corridore, 2020 | | Prevalence of oral disease and treatment types proposed to children affected by Autistic Spectrum Disorder in Pediatric Dentistry: A Systematic Review | | 2008-2018 | | oral health | children (age range NR) | | 13 | | Systematic review | | Autism strongly affects oral health status and from the studies analysed, a clear high incidence of periodontal disease, but also a high caries incidence, was pointed out. | | 0.60 | | | Moderate |
| da Silva, 2017 | | Oral health status of children and young adults with autism spectrum disorders: systematic review and meta-analysis | | 2001-2012 | | oral health | both (2-26 years age range) | | 7 | | Systematic review and meta-analysis | | The pooled prevalence of dental caries and periodontal disease in children and autistic young adults can be considered high, as more than half of assessed autistic individuals presented at least one of these oral conditions. This points to the need of oral health policies for this specific population. | | 0.91 | | | Good |
| Kammer, 2022 | | Prevalence of tooth grinding in children and adolescents with neurodevelopmental disorders: A systematic review and meta-analysis | | 1999-2017 | | tooth grinding | children (age range NR) | | 77 | | Systematic review and meta-analysis | | The pooled prevalence of reported tooth grinding and/or clenching in autistic individuals was 50.4% (95% CI: 35.5–65.4; I²: 93.6%; 7 studies; 703 individuals). The pooled prevalence of clinically observed tooth grinding and/or clenching in autistic individuals was 57.5% (95% CI: 31.6–83.4; I²: 96.6%; 8 studies; 451 individuals). No study included in this review evaluated tooth grinding and/or clenching using polysomnography for a definitive assessment. | | 0.91 | | | Good |
| Lam, 2020 | | Oral health status of children and adolescents with autism spectrum disorder: A systematic review of case-control studies and meta-analysis | | 2001-2017 | | oral health | children and adolescents (age range 4-18 years) | | 16 | | Systematic review and meta-analysis | | 16 studies were included in the qualitative synthesis and 15 were included in quantitative analysis. A weak evidnece showed that autistic children and adolescents had significantly higher prevalence of bruxism. They also had significantly lower salivary pH but the results were not clinically significant that can increase their risks to tooth decay. However, no statistically significant differences in terms of caries prevalence and severity, oral hygiene and periodontal status, prevalence of malocclusion, dental traumatic injuries, as well as salivary flow rate and buffering capacity were found. | | 0.91 | | | Good |
| Pi, 2020 | | A Meta-Analysis of Oral Health Status of Children with Autism | | 2011-2018 | | oral health | children and adolescents (age range 4-16 years) | | 8 | | Systematic review and meta-analysis | | Metaanalysis showed that the mean DMFT index in autistic children was higher than that in healthy children, and the difference was statistically significant {MD = 0.50, 95% CI [0.04–0.96], P<0.00001}. Similarly, plaque index (PI) and gingival index (GI) in autistic children were higher than those in healthy children, and the difference between PI was statistically significant {MD = 0.59, 95%CI [0.36–0.82], P=0.02}, while the difference between GI was not statistically significant {MD = 0.52, 95%CI [0.30–0.75], P=0.08}. But the salivary pH in autistic children was lower than that in healthy children {MD = -0.28, 95%CI [-0.54–-0.02], P = 0.02}, and the difference was statistically significant. | | 0.86 | | | Good |
| Uliana, 2024 | | Autistic individuals have worse oral status than neurotypical controls: a systematic review and meta-analysis of observational studies | | 2001-2023 | | oral health | both (age range NR) | | 42 | | Systematic Review and Meta-analysis | | Autistic individuals had significantly higher severity of dental-caries experience in primary teeth (SMD 0.29, 95%CI 0.02, 0.56), of dental plaque presence (SMD 0.59, 95%CI 0.24, 0.94), and of gingivitis (SMD 0.45, 95%CI 0.02, 0.88). Autistic individuals showed higher probability of occurrence of gingivitis (RR 1.34, 95%CI 1.08, 1.66,), bruxism (RR 4.23, 95%CI 2.32, 7.74), overjet (RR 2.16, 95%CI 1.28, 3.64), overbite (RR 1.62, 95%CI 1.02, 2.59), crossbite (RR 1.48, 95%CI 1.02, 2.13), and openbite (RR 2.37, 95%CI 1.46, 3.85), when compared to neurotypical individuals. Most estimates showed a small effect size with very low certainty of evidence. | | 0.95 | | | Good |
| Dermatitis | | | | | | | | | | | | | | | | | | |
| Billeci, 2015 | | Association Between Atopic Dermatitis and Autism Spectrum Disorders: A Systematic Review | | NR | | atopic dermatitis | both (0-26 years) | | 18 | | systematic review | | Overall, the results of this systematic review seem to reveal an association between autism and  Atopic Dermatitis, suggesting that autistic subjects have an increased risk of presenting with AD compared with typically developing controls, and vice versa. This association is supported by clinical/epidemiological aspects, shared genetic background and common immunological and autoimmune processes. However, the variability in study population and design, and the presence of other risk factors acting as confounding factors, sometimes contribute to inconsistent results. | | 0.70 | | | Good |
| Cheng, 2022 | | Associations of atopic dermatitis with attention deficit/hyperactivity disorder and autism spectrum disorder: a systematic review and meta-analysis | | 2013-2021 | | atopic dermatitis (AD) | both (mean age range 0.71-51 years) | | 24 | | Systematic review and meta-analysis | | Meta-analysis demonstrated significant associations of AD with autism (pooled OR, 1.87; 95% CI, 1.30–2.68). | | 0.95 | | | Good |
| Tsai, 2020 | | Association Between Atopic Dermatitis and Autism Spectrum Disorder: A Systematic Review and Meta-analysis | | 2006-2016 | | atopic dermatitis | children and adolescents (age range 1 month-17 years) | | 16 | | Systematic review and meta-analysis | | A significant correlation between atopic dermatitis and autism was found. | | 0.91 | | | Good |
| Diabetes, asthma and epilepsy | | | | | | | | | | | | | | | | | | |
| Cortese, 2022 | | Association between autism spectrum disorder and diabetes: systematic review and meta-analysis | | 2006-2021 | | diabetes and hypertension | both (0-64 years age range) | | 24 | | Systematic review and meta-analysis | | Despite its limitations, our work suggests that claims on a significant association between autism and diabetes are currently not supported by robust evidence. Future research should focus on moderators that may explain significant associations in subgroups of individuals. Meanwhile, stakeholders and policy makers should be aware that any suggestion of systematically screening diabetes in autistic individuals and vice versa is not grounded on solid evidence. | | 1.00 | | | Good |
| Dhanasekara, 2023 | | Association Between Autism Spectrum Disorders and Cardiometabolic Diseases: A Systematic Review and Meta-analysis | | 2006-2022 | | Diabetes (DM) and hypertension | both (mean age of individuals with autism: 22.8 [range 3.8-72.8] years; mean age of individuals without autism: 31.2 [range 3.8-72.8] years) | | 34 | | Systematic review and meta-analysis | | Results of this systematic review and meta-analysis suggest that autism seems to be associated with an increased risk of DM, dyslipidemia, and atherosclerotic heart disease. Autistic children seem to possess a higher risk of developing DM and hypertension compared with children without autism. Because developing cardiometabolic disease at an early age raises morbidity and health concerns, the need for health care, and mortality, clinicians should vigilantly monitor autistic individuals for early signs of cardiometabolic disease and their complications. | | 0.86 | | | Good |
| Tromans, 2020 | | The prevalence of diabetes in autistic persons: A systematic review | | 2006-2020 | | diabetes | both (age range 0-78 years) | | 19 | | Systematic review | | Of 15 studies that included a non-autistic control group, 9 reported a higher diabetes prevalence among autistic persons, with a statistically significant difference in 4 studies. Studies demonstrating a higher diabetes prevalence in autistic groups had higher average study population sizes and reporting quality ratings. | | 0.90 | | | Good |
| Kaas, 2021 | | Association between childhood asthma and attention deficit hyperactivity or autism spectrum disorders: A systematic review with meta-analysis | | 1995-2019 | | asthma | children (age range 1-19 years) | | 25 | | Systematic review and meta-analysis | | This systematic review with meta-analysis shows no evidence of an association between childhood asthma and autism. | | 0.95 | | | Good |
| Miyazaki, 2015 | | Allergies in Children with Autism Spectrum Disorder: a Systematic Review and Meta-analysis | | 1995-2013 | | allergies, athma, atopic rhinitis | children and adolescents (mean age range 2.5-18 years) | | 10 | | Systematic review and meta-analysis | | A high estimated prevalence of asthma (OR 1.69, 95 % CI 1.11 to 2.59; 2,191 AUTISMchildren) and atopic rhinitis (OR 1.66, 95 % CI 1.49 to 1.85; 1,973 autistic children) were indicated. Rates of food allergy did not show significant differences between groups. Currently, clinical evidence was not found to draw any specific clinical implication. | | 0.94 | | | Good |
| Zheng, 2016 | | Association between Asthma and Autism Spectrum Disorder: A Meta-Analysis | | 2008-2015 | | asthma | both (age range 2-26 years) | | 10 | | Literature review | | The prevalence of asthma in autism was 20.4%, while the prevalence of asthma in controls was 15.4% (P < 0.001). The pooled odds ratio (OR) for the prevalence of asthma in autism in the cross-sectional studies was 1.26 | | 0.92* | | | Good* |
| Liu, 2022 | | Prevalence of epilepsy in autism spectrum disorders: A systematic review and meta-analysis | | 1992-2020 | | epilepsy | both (age range 0-65 years) | | 53 | | Systematic review and meta-analysis | | The updated pooled prevalence of epilepsy in autistic individuals was 10% (95% CI: 6–14). The respective prevalence estimate of epilepsy was 19% (95% CI: 6–35) in the clinical sample-based cross-sectional study, 7% (95% CI: 3–11) in the cohort study, and 9% (95% CI: 5–15) in the population-based cross-sectional study. The pooled prevalence of epilepsy was 7% (95% CI: 4–11) in autistic children and 19% (95% CI: 14–24) in autistic adults. Compared to the school-aged group, the adolescence group (OR: 1.15, 95% CI: 1.06–1.25) and the pre-school group (OR: 1.06, 95% CI: 0.94–1.19) were positively associated with the prevalence of epilepsy. | | 1.00 | | | Good |
| Lukmanji, 2019 | | The co-occurrence of epilepsy and autism: A systematic review | | 1950-2016 | | epilepsy | both (age range 0-82 years) | | 74 | | Systematic review | | The median overall period prevalence of epilepsy in autistic people was 12.1% while the median overall period prevalence of autism in people with epilepsy was 9.0% when including all population types. When excluding studies that investigated patients with syndromic epilepsy or developmental delay, the median overall period prevalence of epilepsy in autistic people was 11.2% while the median overall period prevalence of autism in people with epilepsy was 8.1%. We observed trends for sex as the prevalence of autism in epilepsy was higher in males while the prevalence of epilepsy in autism was higher in females. It is important to interpret these estimates with caution, as there was significant heterogeneity between studies. | | 1.00 | | | Good |
| Sleep | | | | | | | | | | | | | | | | | | |
| Cortese, 2020 | | Sleep Disorders in Children and Adolescents with Autism Spectrum Disorder: Diagnosis, Epidemiology, and Management | | NR | | sleep disorder | children and adolescents (age range NR) | | NR | | Narrative review | | Sleep problems are an important, frequent, and impairing comorbidity in children and autistic adolescents. To date, evidence from RCTs on the short-term treatment of sleep problems in autistic youth is limited, with melatonin being the option with the largest amount of evidence pointing to its efficacy and good tolerability. Evidence is also limited on the treatment in the longer term. An increased body of RCTs is desperately needed on available options, which will then make it possible to pool in comparative meta-analyses allowing the establishment of a hierarchy of treatments in terms of efficacy and tolerability. | | 1.00* | | | Good* |
| Diaz-Roman, 2018 | | Sleep in youth with autism spectrum disorders: systematic review and meta-analysis of subjective and objective studies | | 1984-2018 | | sleep | both (mean age range of autistic individuals: 2.3-16.88 years) | | 47 | | Systematic review and meta-analysis | | Compared with typically developing controls, autistic youth significantly differed in 10/14 subjective parameters and in 7/14 objective sleep parameters. | | 0.95 | | | Good |
| Kim, 2023 | | Correlations between sleep problems, core symptoms, and behavioral problems in children and adolescents with autism spectrum disorder: a systematic review and meta-analysis | | 2005-2022 | | sleep | children (mean age range 4.41-4-14.32 years) | | 22 | | Systematic review and meta-analysis | | Correlations between total sleep problems and total core symptoms (r 0.293 [95% confidence interval − 0.095 to 0.604]), total sleep problems and total behavioral problems (r 0.429 [0.299–0.544]), and total core symptoms and total behavioral problems (r − 0.050 [− 0.177 to 0.079]) were found. Statistically significant correlations between specific components of sleep problems, autism core symptoms, and autism behavioral problems were identified. | | 0.82 | | | Good |
| Liang, 2023 | | Age-Related Differences in Accelerometer-Assessed Physical Activity and Sleep Parameters among Children and Adolescents with and Without Autism Spectrum Disorder: A Meta-Analysis | | 1999-2022 | | sleep and activity | children (age range 5.1 to 16.9 years) | | 28 | | Systematic review and meta-analysis | | Compared with peers without autism, autistic children and adolescentshad a small-to-moderate difference in MVPA (g = −0.450; 95% CI, −0.622 to −0.277), total sleep time (g = −0.332; 95% CI, −0.574 to −0.090), sleep efficiency (g = −0.424; 95% CI, −0.645 to −0.203), and a moderate difference in sleep latency (g = 0.514; 95% CI, 0.351 to 0.677) measured by actigraphy. Autistic children and adolescents with experienced an age-related decline in moderate-to-vigorous physical activity (β = −0.049 [95% CI, −0.097 to −0.001]; P = .045) | | 0.95 | | | Good |
| Lugo, 2020 | | Sleep in adults with autism spectrum disorder and attention deficit/hyperactivity disorder: A systematic review and meta-analysis | | 2003-2019 | | sleep | adults (mean age range 20.6-48.4 years) | | 19 | | Systematic review and meta-analysis | | Autistic participants had pooled prevalence of 58.98% (95% CI [−2.00, 119.45]) for insomnia and of 51.18% (95% CI [34.80, 67.56]) for general sleep disorders. | | 0.77 | | | Good |
| Zaffanello, 2023 | | Sleep Disordered Breathing in Children with Autism Spectrum Disorder: An In-Depth Review of Correlations and Complexities | | 2013-2022 | | sleep disordered breathing | Children and adolescents (age range 2-18 years) | | 7 | | Literature review | | The findings reveal a high incidence of sleep disordered breathing in autistic children, emphasizing the importance of early diagnosis and specialized treatment. Obesity in this population further complicates matters, requiring focused weight management strategies. | | 0.75* | | | Good* |
| Weight and eating | | | | | | | | | | | | | | | | | | |
| Cermak, 2010 | | Food Selectivity and Sensory Sensitivity in Children with Autism Spectrum Disorders | | 1986-2008 | | food selectivity and sensitivity | children (1 month-16.2 years) | | 12 | | Literature review | | The criteria for autism have shifted over time, making it difficult to compare studies and to determine whether subgroups of autistic children are at greater or lesser risk for food selectivity and whether this problem attenuates or persists over time. Moreover, the relation of food selectivity, special diets, and nutritional adequacy needs to be examined. Additional research is needed in studies that include carefully characterized participants so that the phenomenon of food selectivity across the spectrum of autistic disorders can be understood. | | 1.00* | | | Good* |
| Curtin, 2014 | | Obesity in children with autism spectrum disorder | | NR | | obesity | children (age range NR) | | NR | | Literature review | | The prevalence of obesity is at least as high, if not higher, in autistic children compared to other children. Research has documented that atypical antipsychotic medication is a clear risk for weight gain in this clinical population.Obesity and its associated sequelae represent significant threats to independent living, self-care, quality of life, and long-term health outcomes for autistic individuals. | | 0.83* | | | Good* |
| Kahathuduwa, 2019 | | The risk of overweight and obesity in children with autism spectrum disorders: A systematic review and meta-analysis | |  | | Obesity | children (age range 2-18 years; mean age range 2.64-14.30 years) | | 35 | | Systematic review and meta-analysis | | Autistic children had a 41.1% greater risk (P = .018) of development of obesity compared with typically developing children living in same geographical regions. | | 1.00 | | | Good |
| Kahathuduwa, 2022 | | Autism spectrum disorder is associated with an increased risk of development of underweight in children and adolescents: A systematic review and meta-analysis | | 1999-2021 | | weight, underweight | children (mean age range 3.9-14.3 years) | | 26 | | Systematic review and meta-analysis | | Underweight was estimated to have an overall prevalence of 6.5% (95% CI = 5.1–8.2) among autistic children (29 study arms -26 studies; 8960 autistic children). Pooled relative risk of underweight in autistic children was 1.285 (95% CI, 0.788–2.098, p = 0.3152) | | 0.95 | | | Good |
| Li, 2020 | | Global prevalence of obesity, overweight and underweight in children, adolescents and adults with autism spectrum disorder, attention-deficit hyperactivity disorder: A systematic review and meta-analysis | | 1997-2020 | | weight status | both (age range 2-NR years) | | 57 | | Systematic review and meta-analysis | | Pooled estimates of the prevalence of obesity, overweight and underweight were 21.8%, 19.8% and 6.4% in autistic individuals, respectively. An increasing trend in the prevalence ofunhealthy weight was observed from autistic children aged 2 to 5 years to adults (obesity: from 16.7% to 31.3%, overweight: from 16.2% to 27.2%, underweight from5.3% to 8.6%). Population-based studies yielded a lower prevalence of obesity, overweight and underweight than clinic-based studies. | | 1.00 | | | Good |
| Margari, 2020 | | Eating and mealtime behaviors in patients with autism spectrum disorder: Current perspectives | | 2010-2019 | | eating behaviour | both (age range 2-27 years) | | 59 | | Narrative review | | Studies evaluating eating problems in autistic children and adolescents are very heterogeneous and they show methodological differences. Therefore, it is not possible to draw conclusions on the predictive power of eating disturbances when recognized in early childhood. Consequently, establishing the role of eating and mealtime behavior abnormalities as an early marker of autism may be a future direction of research. | | 0.83* | | | Good* |
| Mari-Bauset, 2014 | | Food selectivity in autism spectrum disorders: A systematic review | | 1978-2012 | | food selectivity and sensitivity | children and adolescents (age range 1 month-18 years) | | 25 | | Systematic review | | A lack of standardized definitions of the various food selectivity, intake problems, and autism spectrum disorders makes it difficult to compare the results between studies and is a potential source of bias. However, all the articles reviewed were unanimous that food selectivity is associated with autism but the level of evidence is weak. | | 0.65 | | | Moderate |
| Sammels, 2022 | | Autism Spectrum Disorder and Obesity in Children: A Systematic Review and Meta-Analysis | | 2004-2019 | | obesity | children and adolescent (age range 2-18 years) | | 20 | | Systematic review and meta-analysis | | The pooled prevalence of obesity in autistic children was 17% (95% CI: 13–22). The OR for children with obesity in autistic populations and children with obesity in populations without autism was 1.70 (95% CI: 1.44–1.99). The relative risk of obesity in autistic children compared with control children was 1.58 (95% CI: 1.34–1.86). There were no controlled studies reporting on the prevalence of autism in children with obesity. | | 0.77 | | | Good |
| Zheng, 2017 | | Association among obesity, overweight and autism spectrum disorder: a systematic review and meta-analysis | | 2014-2016 | | Obesity | both (mean age range 2-29.4 years) | | 15 | | Systematic review and meta-analysis | | The prevalence of obesity was significantly higher in autistic individuals than in controls (OR=1.84, 95% confidence interval [CI]: 1.37–2.48, P<0.001). However, the prevalence of overweight in autistic individuals was not significantly diferent from that in controls (OR=1.07, 95% CI: 0.83–1.38, P=0.62). | | 0.59 | | | Moderate |
| Gastrointestinal symptoms | | | | | | | | | | | | | | | | | | |
| Gan, 2023 | | Questionnaire-based analysis of autism spectrum disorders and gastrointestinal symptoms in children and adolescents: a systematic review and meta-analysis | | 2016-2021 | | gastrointestinal symptoms | children and adolescents (mean age range AUTISMpopulation: 6 months-11.8 years; typically developing population: 6 months-11.4 years (NR in all studies) | | 25 | | Systematic review and meta-analysis | | The review showed that autistic children with were more likely to have gastrointestinal symptoms than control children. The prevalence rate was 55%, versus 26% in neurotypical children. Thus, among children with gastrointestinal symptoms, the prevalence rate of autism is approximately twice that of TD children. Sharp mentioned that autistic children are more likely than TD children to have eating issues such as food selectivity, food refusal, and poor oral intake, which indicates that autistic children have feeding problem | | 0.91 | | | Good |
| Kim, 2022 | | Association between autism spectrum disorder and inflammatory bowel disease: A systematic review and meta-analysis | | 2012-2019 | | IBS | children (mean age range 12.9-17.5 years) | | 6 | | Systematic review and meta-analysis | | Autism was significantly associated with subsequent incident IBD (any IBD,OR=1.66, 95% confidence interval[CI]=1.25–2.21,p< 0.001; ulcerative colitis,OR=1.91, 95%CI=1.41–2.6,p< 0.001; Crohn’s disease, OR=1.47, 95%CI=1.15–1.88,p=0.002). Autism and IBD were also associated regardless of temporal sequence of diagnosis (any IBD, OR=1.57, 95%CI=1.28–1.93,p< 0.001; ulcerative colitis, OR=1.7, 95%CI=1.36–2.12,p< 0.001; Crohn’s disease,OR=1.37, 95%CI=1.12–1.69,p=0.003) | | 0.82 | | | Good |
| Kittana, 2023 | | Nutritional Status and Feeding Behavior of Children with Autism Spectrum Disorder in the Middle East and North Africa Region: A Systematic Review | | 2010-2020 | | Nutritional Status and Feeding Behavior | both (age range 3-27 years) | | 43 | | Systematic Review | | Both overweight and underweight were common in autism, although not consistently different than typically developing children. Nutrient inadequacies of energy, protein, omega-3, and others; deficiencies in serum iron indicators and calcium, as well as vitamins B12, B9, and D levels; and higher levels of homocysteine and omega-6/omega-3 ratios were reported. Feeding behaviour problems were also common in autism. | | 0.95 | | | Good |
| Lanyi, 2022 | | Abdominal Pain in Children and Adolescents with Autism Spectrum Disorder: a Systematic Review | | 2003-2014 | | gastrointestinal symptoms and abdominal pain | children and adolescents (age range 1 month-19 years) | | 13 | | Systematic review | | There is strong evidence in support of the high prevalence of abdominal pain in autistic children and adolescents compared to TD peers, and there are a number of adverse psychological and behavioral associations with this symptom. | | 0.85 | | | Good |
| Lasheras, 2023 | | Prevalence of gastrointestinal symptoms in autism spectrum disorder: A meta-analysis | | 2013-2021 | | gastrointestinal symptoms | children and adolescents (age range 2.5-17 years) | | 8 | | Systematic review and meta-analysis | | The prevalence of GI symptoms ranged between 0% and 69%, with an estimated general prevalence of 33% (95% CI, 13%–57%), higher than that reported by a previous meta-analysis for the general paediatric population. | | 0.68 | | | Moderate |
| Leader, 2022 | | Gastrointestinal Symptoms in Autism Spectrum Disorder: A Systematic Review | | NR | | gastrointestinal symptoms (GIS) | children and adolescents (age range 6 months-18 years) | | 30 | | Systematic review | | The review found that GIS were common and that there was contradictory evidence concerning their relationship with co-occurring conditions. It also identified evidence of some causal relationships that support the existence of the gut–immune–brain pathways. | | 0.80 | | | Good |
| Li, 2021 | | Association of food hypersensitivity in children with the risk of autism spectrum disorder: a meta-analysis | | 1995-2019 | | food hypersensitivity | children (age range NR) | | 12 | | Systematic review and meta-analysis | | A significant association was observed between food hypersensitivity and the risk of autism (OR = 2.792, 95% CI: 2.081–3.746). The risk of autism among girls and subjects younger than 12 with food hypersensitivity may be greater than that among boys and those older than 12. The results of sensitivity analysis and publication bias analysis show that the association is relatively stable. | | 1.00 | | | Good |
| Lima, 2024 | | Autism in patients with eosinophilic gastrointestinal disease: A systematic review with meta-analysis | | 2016-2022 | | Eosinophilic gastrointestinal disease EGID) | children (median age range 6.5-8.5 years) | | 6 | | Systematic Review and Meta-analysis | | The result of the single-arm meta-analysis showed an overall prevalence of autism in the population with EGID of 21.59% (95% CI: 10.73–38.67). There was an association between EGID and autism (OR: 3.44; 95% CI: 1.25–2.21), also significant when restricted only to EoE (OR: 3.70; 95% CI: 2.71–5.70). | | 0.73 | | | Good |
| McElhanon, 2014 | | Gastrointestinal symptoms in autism spectrum disorder: a meta-analysis | | 2000-2010? | | gastrointestinal symptoms | children (mean age range 50.8-140.5 months) (NR in all studies) | | 15 | | Systematic review and meta-analysis | | Autistic children experience significantly more general GI symptoms than comparison groups, with a standardized mean difference of 0.82 (0.24) and a corresponding odds ratio (OR) of 4.42 (95% CI,1.90–10.28). Analysis also indicated higher rates of diarrhoea (OR, 3.63;95% CI, 1.82–7.23), constipation (OR, 3.86; 95% CI, 2.23–6.71), andabdominal pain (OR, 2.45; 95% CI, 1.19–5.07). Identified studies involved high methodological variability and lack of comprehensive data prohibited analysis of GI pathophysiologies (eg, gastro-esophageal reflux) typically associated with organic etiologies, limiting conclusions about the underpinnings of the observed association. | | 0.73 | | | Good |
| Pinto-silvia, 2024 | | Feeding problems in children with autism spectrum disorders: a systematic review | | 2010-2018 | | feeding problems | children (age range NR) | | 45 | | Systematic review | | Autistic children seem to have a major predisposition to present with atypical feeding. It is particularly marked by limitations in food repertoire and by the presence of mealtime behavioural problems related to their relationship with food or mealtime routines. | | 0.80 | | | Good |
| Sharp, 2013 | | Feeding problems and nutrient intake in children with autism spectrum disorders: a meta-analysis and comprehensive review of the literature | | 1982-2011 | | feeding problems | children (age range 6-127 months) | | 17 | | Systematic review and meta-analysis | | Results indicated autistic children experience significantly more feeding problems versus peers, with an overall SMD of 0.89 (0.08) and a corresponding OR of 5.11, 95 % CI 3.74–6.97. Nutrient analyses indicated significantly lower intake of calcium (SMD: −0.65 [0.29]; OR: 0.31, 95 % CI 0.11–0.85) and protein (SMD: −0.58 [0.25]; OR: 0.35, 95 % CI: 0.14–0.56) in autism. | | 0.82 | | | Good |
| Quan, 2019 | | Association Between Celiac Disease and Autism Spectrum Disorder: A Systematic Review | | 1997-2017 | | celiac disease | children and adolescents (age range 0-18 years) | | 17 | | Systematic review | | The null findings were reported in most studies examining the co-occurrence of celiac disease and autism may be a consequence of systematic and/or random error. Nevertheless, a limited number of higher-quality studies with large sample sizes suggest a potential association between these 2 conditions cannot be excluded. | | 0.90 | | | Good |
| Wang, 2021 | | Association between Autism Spectrum Disorder and Food Allergy: A Systematic Review and Meta-analysis | | 2012-2019 | | food allergy | both (age range 0-26 years) | | 14 | | Systematic review and meta-analysis | | The pooled prevalence of food allergy (FA) in autistic individuals was 13% (95% confidence interval (CI): 0.10–0.17), while the pooled prevalence of FA in controls was 5% (95% CI: 0.04–0.07). The pooled OR for FA in autistic individuals was 2.45 (95% CI: 2.25–2.67). The prevalence of autism in individuals with FA was 3.5% (95% CI: 0.029–0.042), while the prevalence of autism in the control group was 1.6% (95% CI: 0.015–0.017).The OR for autism in individuals with FA was 1.95 (95% CI:1.73–2.19). | | 0.86 | | | Good |
| Wang, 2022 | | Global prevalence of autism spectrum disorder and its gastrointestinal symptoms: A systematic review and meta-analysis | | 2001-2022 | | gastrointestinal symptoms | both (age range 0-72 years) | | 75 | | Systematic review and meta-analysis | | The global pooled prevalence of autism was 98/10,000 (95% confidence interval, 95%CI: 81/10,000–118/10,000, I2 = 99.99%, p < 0.001), with 48.67% (95%CI: 43.50 −53.86) of autistic individuals reporting gastrointestinal symptoms. | | 0.95 | | | Good |
| Mortality | | | | | | | | | | | | | | | | | | |
| Forsyth, 2023 | | All-cause and cause-specific mortality in people with autism spectrum disorder: A systematic review | | 1998-2022 | | mortality |  | | 26 | | Systematic Review | | 25 studies reported an increased risk of mortality for autistic people. Out of 21 studies reporting the relevant statistics, 15 found autistic individuals to have at least a two times higher risk of dying when compared to the general population. 11 studies suggested that autistic females were at an even greater risk of death when compared to their male counterparts. The most common causes of deaths were from external causes (particularly suicide) and [neurological disorders](https://www.sciencedirect.com/topics/psychology/neurological-disorder). | | 0.95 | | | Good |
| Woolfenden, 2012 | | A systematic review of two outcomes in autism spectrum disorder - epilepsy and mortality | | 1992-2010 | | Mortality | children and adolescents (mean age range 2.6-16.9 years) | | 21 | | Systematic review | | The pooled estimate for the percentage of participants with epilepsy was 1.8% (95% CI 0.4–9.4%) in studies in which the majority did not have an intellectual disability and the mean age was <12 years at follow-up, and 23.7% (95% CI 17.5–30.5%) in studies in which the majority did have an intellectual disability and the mean age at follow-up was more than 12 years. The pooled estimate for the standardized mortality ratio was 2.8 (95% CI 1.8–4.2). | | 0.90 | | | Good |
| Others | | | | | | | | | | | | | | | | | | |
| Beers, 2014 | | Autism and peripheral hearing loss: a systematic review | | NR | | hearing loss | NR | | 33 | | systematic review | | Controversy exists in the literature regarding the prevalence of hearing impairment among autistic individuals. In cases where autism and hearing impairment co-exist, diagnosis of one condition often leads to a delay in diagnosing the other. | | 0.85 | | | Good |
| Gu, 2023 | | The Association Between Congenital Heart Disease and Autism Spectrum Disorder: A Systematic Review and Meta-Analysis | | 2002-2020 | | Congenital heart disease (CHD) | both (mean age range 2.21-58 years) | | 24 | | Systematic Review and Meta-analysis | | Seven of 24 studies were eligible for the meta-analysis, which included information on a total of 250,611 subjects (3984 CHD, 9829 autism, and 236,798 controls). The summary estimate indicated that having CHD is associated with almost double the odds of autism compared with patients without CHD (OR 1.99, 95% CI 1.77–2.24, p < 0.01). Early developmental delay, perinatal factors, and genetics were potential risk factors and etiologies for the onset of autism symptoms in CHD patients. | | 0.77 | | | Good |
| Melo, 2020 | | Prevalence and determinants of motor stereotypies in autism spectrum disorder: A systematic review and meta-analysis | | 1976-2015 | | stereotypies (motor movement) | both (average age range 1-52 years) | | 37 | | Systematic review and meta-analysis | | The most frequent determinants associated with a higher number of stereotypies in autism were a younger age, lower intelligence quotient, and a greater severity of autism. Moreover, gender did not seem to influence the prevalence of stereotypies. Meta-analytic analysis showed that lower IQ and autism diagnosis (independent of IQ) are associated with a higher prevalence of motor stereotypies (odds ratio = 2.5 and 4.7, respectively). | | 0.91 | | | Good |
| Niemczyk, 2018 | | Incontinence in autism spectrum disorder: a systematic review | | 1987-2015 | | incontinence | both (age range 2-38 years) | | 33 | | Systematic review | | The literature implies a higher prevalence of incontinence in autistic children with compared to typically developing children. Associations of incontinence in autism with psychopathological symptoms were found and vice versa. All types of incontinence including nocturnal enuresis, daytime urinary incontinence, | | 0.70 | | | Good |
| Pan, 2021 | | Neurological disorders in autism: A systematic review and meta-analysis | | 1992-2019 | | neurological disorders review | both (age range NR) | | 79 | | Systematic review and meta-analysis | | Autistic individuals were significantly more likely than the general population to exhibit epilepsy, macrocephaly, hydrocephalus, cerebral palsy, migraine/headache, and congenital abnormalities of the nervous system, with prevalence estimates ranging from 1.1% (0%–3.3%; hydrocephalus) to 14.2% (11.3%–17.2%; epilepsy). Epilepsy was also more common in autism than in ADHD (odds ratio [95% confidence interval] = 4.06 [2.81–5.88]). | | 1.00 | | | Good |
| Perna, 2023 | | Association between Autism Spectrum Disorder (ASD) and vision problems. A systematic review and meta-analysis | | 2005-2021 | | vision problem | NR | | 49 | | Systematic review and meta-analysis | | Increased prevalence of strabismus (OR = 4.72 [95% CI: 4.60, 4.85]) in people with versus those without autism. Evidence of increased accommodation deficits (Hedge’s g = 0.68 [CI: 0.28, 1.08]), reduced peripheral vision (−0.82 [CI: −1.32, −0.33]), reduced stereoacuity (0.73 [CI: −1.14, −0.31]), increased color discrimination difficulties (0.69 [CI: 0.27,1.10]), reduced contrast sensitivity (0.45 [CI: −0.60, −0.30]) was also found | | 0.86 | | | Good |
| Vaquerizo-Serrano, 2022 | | Catatonia in autism spectrum disorders: A systematic review and meta-analysis | | 2000-2017 | | catatonia | both (mean age range 2.8-27.6 years) | | 12 | | Systematic review and meta-analysis | | 10.4% (5.8–18.0 95%CI) of autistic individuals have catatonia. Motor disturbances were common in autistic subjects with catatonia. | | 0.86 | | | Good |
| Woolfenden, 2012 | | A systematic review of two outcomes in autism spectrum disorder - epilepsy and mortality | | 1992-2010 | | epilepsy and mortality | children and adolescents (mean age range 2.6-16.9 years) | | 21 | | Systematic reivew | | The pooled estimate for the percentage of participants with epilepsy was 1.8% (95% CI 0.4–9.4%) in studies in which the majority did not have an intellectual disability and the mean age was <12 years at follow-up, and 23.7% (95% CI 17.5–30.5%) in studies in which the majority did have an intellectual disability and the mean age at follow-up was more than 12 years. The pooled estimate for the standardized mortality ratio was 2.8 (95% CI 1.8–4.2). | | 0.90 | | | Good |
| Social and lifestyle | | | | | | | | | | | | | | | | | | |
| Multiple factors systematic reviews | | | | | | | | | | | | | | | | | | |
| Howlin, 2017 | | Autism spectrum disorder: Outcomes in adulthood. | | 2015-2016 | | adult outcomes | adults | | 43 | | Literature review | | Overall outcomes in terms of jobs, relationship, independent living and mental health are poorer for autistic adults than for same age peers | | 1.00* | | | Good* |
| Micai, 2023 | | Prevalence of co-occurring conditions in children and adults with autism spectrum disorder: A systematic review and meta-analysis | | 1984-2021 | | co-occuring conditions | both (mean age range 0.9-67 years) | | 340 | | Systematic review and meta-analysis | | Among the mental health/psychiatric co-occuring conditions (CCs), the most frequently reported CCs, with their point pooled prevalence estimates, were developmental coordination disorder, sleep-wake problem, ADHD, anxiety disorder, ID (point and lifetime prevalence pooled together), feeding and eating disorder, disruptive behaviour, somatic symptom and related disorders, and sleepwake disorder. | | 0.91 | | | Good |
| Matson, 2013 | | Comorbidity and autism: Trends, topics and future directions | | NR | | comorbidities | both (age range NR) | | 449 | | Literature review | | High rates of comorbidity between personality and anxiety disorder were found. Similarly, ADHD symptoms have been commonly reported among persons with bipolar disorder. A host of other conditions have also been reported as co-occurring. | | 0.67* | | | Moderate* |
| Body image and identity | | | | | | | | | | | | | | | | | | |
| Bouzy, 2023 | | Transidentities and autism spectrum disorder: A systematic review | | 2010-2022 | | trans identity and gender incongruency | both (3.22-75 years age range) | | 77 | | Systematic review | | Regarding the frequencies of autism in trans populations and of transidentity in people with autism, the results are quite heterogeneous but show an overrepresentation of this cooccurrence. | | 0.40 | | | Poor |
| Longhurst, 2023 | | Body image and autism: A scoping review | | 2016-2022 body image | | body image | both (age range 11-75 years) | | 8 | | Scoping review | | Findings suggest there is a significant relationship between indices of negative body image (e.g., body dissatisfaction) and autistic traits. This review also suggests that autistic individuals experience both negative and positive aspects of body image, informed by autismspecific factors such as differences in social communication, information processing, and sensory sensitivities. The extant research is, however, limited to either neurotypical samples or autistic individuals in clinical contexts (e.g., eating disorders, weight management). This review identified a paucity of robust research investigating body image and | | 1.00* | | | Good* |
| Glidden, 2016 | | Gender Dysphoria and Autism Spectrum Disorder: A Systematic Review of the Literature | | 2015-2021 | | gender dysphoria | both (age range NR) | | 19 | | Systematic review | | The literature investigating autism in children and adolescents with gender dysphoria showed a higher prevalence rate of autism compared with the general population. There is a limited amount of research in adults. Only one study showed that adults attending services for gender dysphoria had increased autism scores. Another study showed a larger proportion of adults with atypical gender identity and autism. | | 0.60 | | | Moderate |
| Bullying, victimisation and violence | | | | | | | | | | | | | | | | | | |
| Dike, 2023 | | A Systematic Review of Sexual Violence Among Autistic Individuals | | 2005-2021 | | sexual violence | both (age range NR) | | 22 | | Systematic review | | It is possible that autistic individuals may be particularly vulnerable to sexual violence, or more able to report sexual violence, as they move into adulthood. | | 0.75 | | | Good |
| Gibbs, 2024 | | Prevalence and Risk Factors Associated with Interpersonal Violence Reported by Autistic Adults: A Systematic Review | | 2016-2023 | | Interpersonal violance | adolescents and adults (age range 18-64 years) | | 16 | | Systematic Review | | Overall results indicated that rates of interpersonal violence may be higher than in the general population. Rates of sexual violence for autistic adults ranged between 7.7% and 64%, physical violence between 8 and 60%, and emotional abuse between 22 and 70%. | | 0.95 | | | Good |
| Maiano, 2016 | | Prevalence of School Bullying Among Youth with Autism Spectrum Disorders: A Systematic Review and Meta-Analysis | | 2008-2015 | | bullying | children and adolescents (age range 9.25- 15.08 years) (NR in all studies) | | 17 | | Systematic review and meta-analysis | | The resulting pooled prevalence estimate for general school bullying perpetration, victimization and both was 10%, 44%, and 16%, respectively. Pooled prevalence was also estimated for physical, verbal, and relational school victimization and was 33%, 50%, and 31%, respectively. | | 0.95 | | | Good |
| Park, 2020 | | Prevalence of and Factors Associated with School Bullying in Students with Autism Spectrum Disorder: A Cross-Cultural Meta-Analysis | | 1998-2018 | | school bullying | children and adolescents (age range 5-22 years) | | 34 | | Systematic review and meta-analysis | | Pooled prevalence estimates for victimization, perpetration, and perpetration-victimization in general were 67%, 29%, and 14%, respectively. The risk of victimization in autistic students was significantly higher than that in typically developing students and students with other disabilities. Further, deficits in social interaction and communication, externalizing symptoms, internalizing symptoms, and integrated inclusive school settings were related to higher victimization, and externalizing symptoms were related to higher perpetration. | | 1.00 | | | Good |
| Sreckovic, 2014 | | Victimization of students with autism spectrum disorder: A review of prevalence and risk factors | | 2002-2013 | | victimization | children and adolescents (age range 4-21 years) | | 21 | | Systematic literature review | | Prevalence studies suggest autictic students are frequent victims of bullying with victimization rates ranging by study methodology. Studies reporting factors related to the victimization of autistic students include individual (i.e., characteristics of autism, social vulnerability, behavior problems, disability, race, academic achievement, and age of student) and contextual (i.e., educational setting, school transportation, parental mental health, parental engagement and confidence, family socioeconomic status, and social support from peers and friendship) factors. | | 1.00* | | | Good* |
| Trundle, 2023 | | Prevalence of Victimisation in Autistic Individuals: A Systematic Review and Meta-Analysis | | 2005-2019 | | victimisation | both (age range 1-57 years) | | 34 | | Systematic review and meta-analysis | | Meta-analysis found a pooled prevalence rate of victimisation of 44% in autistic individuals. The pooled prevalence rates for bullying was 47%, 16% for child abuse, 40% for sexual victimisation, 13% for cyberbullying, and 84% for multiple forms of victimisation in autistic individuals. | | 0.82 | | | Good |
| Gaming | | | | | | | | | | | | | | | | | | |
| Craig, 2021 | | A systematic review of problematic video-game use in people with Autism Spectrum Disorders | | 212-2020 | | game usage | Both (9.8 - 20.54) | | 12 | | systematic review | | Twelve articles were included in this systematic review. The majority of them indicate that autistic children, adolescents and adults may be at greater risk of PVG use than youths without autism. Findings also suggested that several internal (sex, attention and oppositional behaviour problems) and external factors (social aspects, access and time spent playing video, parental rules, and game genre) were significant predictors of problematic video game use in autistic people. In addition, this review highlights the paucity of the literature on the consequences and individual effects of excessive video gaming in autistic people . | | 0.85 | | | Good |
| Murray, 2022 | | Autism, problematic internet use and gaming disorder: A systematic review | | 2012-2020 | | gaming and problematic internet use | both (age range 4-79 years) | | 21 | | Systematic review | | The majority of the studies found positive associations between problematic internet use (PIU) and subclinical autistic-like traits with weak and moderate effect sizes and between PIU and autismwith varying effect sizes. Additionally, autistic individuals were more likely to exhibit symptoms ofgaming disorder (GD) with moderate and strong effect sizes. | | 0.90 | | | Good |
| Offending and criminality | | | | | | | | | | | | | | | | | | |
| Allely, 2016 | | Sexual offending and autism spectrum disorders | | 2008-2014 | | sexual offending | both (14-59 years) | | 14 | | systematic review | | The papers identified in this review highlight a relatively modest number of studies which have found a number of features of autism which can contribute to sexual offending in a small minority of autistic individuals. Some of these symptoms include: obsession or preoccupation with certain things (e.g. women’s underwear), failure to conform to social conventions, impaired ToM, impaired ability to decode language and social gestures and a limited repertoire of appropriate behaviour. It is important to recognise the potential impact of these features in a sexual offender with autism, particularly in the case of an adolescent who has committed sexually inappropriate behaviour, as being labelled a sexual offender or “deviant” will only act to further exacerbate their existing impaired social and emotional abilities | | 0.70 | | | Good |
| King, 2014 | | A systematic review of people with autism spectrum disorder and the criminal justice system | | 1994-2012 | | Criminality | both (age range 6-30 years - NR in all studies) | | 22 | | Systematic review | | The results from the seven existing studies that focused on prevalence rates of autismin parts of the CJS .all found overall rates above 1 %, at least in the more able Asperger groups, so it seems likely that autistic people are somewhat over-represented in the CJS. The association is weak and not conclusive. | | 0.45 | | | Moderate |
| Margari, 2024 | | Autism spectrum disorder (ASD) and sexual offending: A systematic review | | 1998-2020 | | sexual offence | both (age range NR) | | 19 | | Systematic review | | Autistic individuals are not at higher risk of committing crimes. | | 0.90 | | | Good |
| Rutten, 2017 | | Autism in adult and juvenile delinquents: A literature review | | NR | | delinquency | both (mean age range 10.7-42 years) | | 12 | | Literature review | | The prevalence of delinquency in the autistic population varied from 5 to 26%, whilst autism was found in 2–18% of the forensic populations studied. The reported prevalence of autism in delinquents and of offending in autistic people varied widely. This might be due to the use of different diagnostic instruments, the diversity of the samples, the high rate of comorbid psychiatric disorders and the various types of offending behaviour. | | 1.00* | | | Good* |
| Quality of life | | | | | | | | | | | | | | | | | | |
| Ayres, 2018 | | A systematic review of quality of life of adults on the autism spectrum. | | 2006-2016 | | quality of life (QoL) | adults (18-55 years old with one study including participants up to 83 years) | | 14 | | systematic review | | QoL for autistic adults is lower than that of typically developing adults, when measured with tools designed for the general population. There are no comprehensive Autism-specific QoL measurement tools validated for use with representative samples of adults on the autism spectrum. There is a pressing need to develop robust measures of QoL in autistic adults. | | 1.00 | | | Good |
| van Heijst, 2015 | | Quality of life in autism across the lifespan: a meta-analysis | | 2004-2012 | | QoL | adults (age range 53-83 years) | | 10 | | meta-analysis | | The meta-analysis showed that quality of life is lower for autistic people compared to people without autism, and that the mean effect is large (Cohen’s d = −0.96). | | 0.73 | | | Good |
| Relationship and connection | | | | | | | | | | | | | | | | | | |
| Hancock, 2017 | | Socio-sexual functioning in autism spectrum disorder: A systematic review and meta-analyses of existing literature | | 2005-2015 | | socio-sexual functioning | both (age range 10-36 years) | | 6 | | Systematic review and meta-analysis | | Autistic individuals have greater difficultly adhering to privacy norms, engage in less social behaviour, are described as engaging in less appropriate sexual behaviour, have greater concerns about themselves, and receive less sexual health education. Having fewer opportunities for appropriate informal and formal sexual health education leaves them at a double disadvantage from others who are receiving this information from both of these avenues. | | 0.41 | | | Poor |
| Hymas, 2022 | | Loneliness in Autism and Its Association with Anxiety and Depression: A Systematic Review with Meta-Analyses | | 2000-2021 | | loneliness | both (AUTISMpopulation: mean age range 9.90-41.9 years; NT population: mean age range9.30-43.7 years) | | 39 | | Systematic review and meta-analysis | | All the included studies found increased loneliness in autistic people compared to neurotypical samples (significant difference in 21 studies) - the combined weighted effect size for this difference was large (Hedges’ g = 0.89). | | 1.00 | | | Good |
| Mamas, 2021 | | Social participation of students with autism spectrum disorder in general education settings | | 2005-2017 | | school social participation | children and adolescents (age range NR) | | 24 | | Systematic review | | Overall, the results are mixed. On the whole, autistic students have been found to maintain a lower social participation status within their schools, but some encouraging findings have also been reported, especially at the elementary school level. In the secondary school level, autistic students were found to face more challenges in regards to peer social participation and interactions. Implications for special and inclusive practice and research are being discussed. | | 0.60 | | | Moderate |
| Mendelson, 2016 | | Friendship in school-age boys with autism spectrum disorders: A meta-analytic summary and developmental, process-based model | | 2000-2014 | | friendships | only boys (age range 8-12 years) | | 18 | | Systematic review and meta-analysis | | This review found that, school-aged autistic boys can and do  make friends. However, they report fewer and lower quality  friendships and receive lower sociometric ratings from peers.  These differences in friendship number and quality, may impact the ability of autistic boys to reap the benefits of reciprocal friendship. While TD boys derive benefits including improved psychosocial functioning and improved awareness of social norms, findings linking processes of friendship to its outcomes among autistic individuals have not been clear-cut. | | 0.73 | | | Good |
| Pecora, 2016 | | Sexuality in High-Functioning Autism: A Systematic Review and Meta-analysis | | 2005-2015 | | sexuality | both (age range 10-39 years) | | 27 | | Systematic review and meta-analysis | | Pooled data from 9 studies revealed that autistic individuals have significantly lower levels of sexual knowledge (d = −1.11; p < .001; 95 % CI −1.44 to −0.88; k = 4); have less social contact and engage in fewer social behaviours (d = 0.12; p < .01; 95 % CI −1.07 to 1.30; k = 4); engage in more inappropriate sexual behaviours (d = 0.58; p < 0.05; CI 0.07–1.09; 3); and are the subject of greater parental concern (d = 1.16; p < .05; 95 % CI 0.15–2.16; k = 3) than TD individuals. | | 0.77 | | | Good |
| Petrina, 2014 | | The nature of friendship in children with autism spectrum disorders: A systematic review | | 2000-2013 | | friendship | children and adolescents (age range 4-18 years) (NR in all stuides) | | 24 | | Systematic review | | The results of this review indicate important differences in the manifestation of friendships in autistic individuals as compared to typical children. While there is consistent evidence for several topographical differences in friendship characteristics, a number of gaps in our knowledge are evident. These include limited data on children who have intellectual disability, and on the perspective of nominated friends as well as circumscribed data on satisfaction with friendship relationships. | | 0.78 | | | Good |
| Miscellaneous | | | | | | | | | | | | | | | | | | |
| Schwartzman, 2022 | | Depression and Employment Outcomes in Autistic Adults: A Systematic Review | | 2011-2021 | | depression and unemployment | adults (mean age range 20.4–61.5 years) | | 21 | | Systematic review | | Clinically-significant depressive symptoms were frequently reported by autistic adults (within-sample mean of 44% autistic adults with elevated scores), while active employment was less frequently reported (26–55% of adults employed in some capacity). Severe depression and/or suicidality were more common in autistic adults than non-autistic adults, while full-time employment was less common in autistic adults. Of the 10 studies that empirically tested associations between depression and employment, findings suggest that employment does not predict depression and/or suicidality. | | 0.60 | | | Moderate |
| Nordin, 2023 | | School absenteeism in autistic children and adolescents: A scoping review | | 1991-2022 | | school absenteeism | children and adolescents (age range 2-21 years) | | 46 reports (from 42 separate studies) | | Scoping review | | Autistic children and adolescents were absent from school more often than their non-autistic peers, which partly was attributable to co-occurring conditions. Bullying also emerged as a potential risk factor. | | 1.00* | | | Good* |
| Perkins, 2012 | | Into the unknown: Aging with autism spectrum disorders | | NR | | ageing with autism, mental health, physical health, quality of life | NR | | NR | | Literature review | | The emerging literature indicates that autism behavioural characteristics appear changeable across the lifespan, and comorbidities, including epilepsy and ID, and mental health issues, including anxiety and depression, can reduce quality of life. Social isolation, restriction in both vocational/employment opportunities, and residential options have all been noted. | | 1.00* | | | Good* |
| Silvi, 2018 | | A Literature Review of the Likely Effects of Autism Spectrum Disorder on Adolescent Driving Abilities | | 2010-2017 | | driving | adolescents and adults (mean age range 15.4-34.3 years) | | 9 | | Systematic literature review | | The literature revealed that autistic drivers (particularly males) were less likely to identify social hazards (e.g., pedestrians), had slower reaction times, more tactical driving difficulties, reported more traffic crashes, citations and intentional driving violations, and had poorer situation awareness skills than drivers without autism. | | 0.92* | | | Good* |

*Quality rating for non-systematic review was done with SANRA assessment tool
